# On the privacy of mental health apps: An empirical investigation and its implications for app development

**DOI:** 10.1007/s10664-022-10236-0

**Published:** 2022-11-08

**Authors:** Leonardo Horn Iwaya, M. Ali Babar, Awais Rashid, Chamila Wijayarathna

**Affiliations:** 1 Centre for Research on Engineering Software Technologies, The University of Adelaide, Adelaide, SA 5005 Australia; 2 Cyber Security Cooperative Research Centre (CSCRC), Joondalup, Australia; 3 Privacy and Security (PriSec), Department of Mathematics and Computer Science, Karlstad University, Karlstad, Sweden; 4 Bristol Cyber Security Group, Department of Computer Science, University of Bristol, Bristol, UK; 5 REPHRAIN: National Research Centre on Privacy, Harm Reduction and Adversarial Influence Online, Bristol, UK

**Keywords:** Privacy, Security, Mobile health, Mental health apps, Privacy by design, Android, Empirical study

## Abstract

An increasing number of mental health services are now offered through mobile health (mHealth) systems, such as in mobile applications (apps). Although there is an unprecedented growth in the adoption of mental health services, partly due to the COVID-19 pandemic, concerns about data privacy risks due to security breaches are also increasing. Whilst some studies have analyzed mHealth apps from different angles, including security, there is relatively little evidence for data privacy issues that may exist in mHealth apps used for mental health services, whose recipients can be particularly vulnerable. This paper reports an empirical study aimed at systematically identifying and understanding data privacy incorporated in mental health apps. We analyzed 27 top-ranked mental health apps from Google Play Store. Our methodology enabled us to perform an in-depth privacy analysis of the apps, covering static and dynamic analysis, data sharing behaviour, server-side tests, privacy impact assessment requests, and privacy policy evaluation. Furthermore, we mapped the findings to the LINDDUN threat taxonomy, describing how threats manifest on the studied apps. The findings reveal important data privacy issues such as unnecessary permissions, insecure cryptography implementations, and leaks of personal data and credentials in logs and web requests. There is also a high risk of user profiling as the apps’ development do not provide foolproof mechanisms against linkability, detectability and identifiability. Data sharing among 3rd-parties and advertisers in the current apps’ ecosystem aggravates this situation. Based on the empirical findings of this study, we provide recommendations to be considered by different stakeholders of mHealth apps in general and apps developers in particular. We conclude that while developers ought to be more knowledgeable in considering and addressing privacy issues, users and health professionals can also play a role by demanding privacy-friendly apps.

**Supplementary Information:**

The online version contains supplementary material available at 10.1007/s10664-022-10236-0.

## Introduction

The ongoing COVID-19 pandemic has dramatically increased the number of mental health support services provided using applications developed for mobile devices. Such applications are called mental health apps, a subcategory of mobile health (mHealth) systems. Examples are chatbots (e.g., Wysa and Woebot) and text-a-therapist platforms (e.g., TalkSpace and BetterHelp) that can be readily downloaded from apps stores, e.g., iOS or Android, and used for seeking and/or providing help for mental health well-being (?echalliance; Heilweil 2020). Even before the COVID-19 pandemic, these apps have made the provision of mental health services more accessible to the people in need, by lowering cost, eliminating traveling, saving time and reducing the fear of social stigma/embarrassment attached to psychological treatment (Bakker et al. 2016; Price et al. 2014). Furthermore, mental health apps increase the availability of mental health services (“anywhere and anytime”) to users and provide additional functionalities such as real-time monitoring of users (Donker et al. 2013). Research also shows that mental health apps improve users’ autonomy and increase self-awareness and self-efficacy (Prentice and Dobson 2014) leading to better health outcomes.

On the other hand, studies on the security of mHealth apps, in general, have shown that many apps are insecure, threatening the privacy of millions of users (Papageorgiou et al. 2018). Insecure apps can be the prime targets of cyber attackers since personal health information is of great value for cyber-criminals (IBM 2020). There is also increasing evidence pointing to a widespread lack of security knowledge among mHealth developers, which is usually linked to different issues, such as insufficient security guidelines, tight budgets and deadlines, lack of security testing, and so on Aljedaani et al. (2020) and Aljedaani et al. (2021). App developers also heavily rely on a range of SDKs for analytics and advertising, exacerbating the risks of data linkage, detectability, and re-identification of users in such ecosystems (Solomos et al. 2019).

The real or perceived security risks leading to data privacy compromises are particularly concerning for mental health apps because they deal with highly sensitive data, in contrast to other general mHealth apps, e.g., for fitness and wellness. The stigma around mental illnesses also increases the potentially negative impacts on users in case of privacy violations. For instance, the mere link of users to a given app can reveal that they might be having some psychological problems (e.g., anxiety, depression, or other mental health conditions), which may make mental health apps users feel more vulnerable and fragile.

The above-mentioned mHealth apps’ data privacy concerns warrant evidence-based inquiries for improved understanding and actionable measures as there is a paucity of empirical research on understanding the full range of privacy threats that manifest in mental health apps; the existing research has only focused on privacy policy analysis (O’Loughlin et al. 2019; Powell et al. 2018; Robillard et al. 2019; Rosenfeld et al. 2017), or 3rd-party data sharing (Huckvale et al. 2019). Hence, it is important to systematically identify and understand the data privacy problems that may exist in mHealth apps as such a body of knowledge can better inform the stakeholders in general and apps developers in particular.

In this study, we specifically focus on the subgroup of mHealth apps designed for mental health and psychological support. This study was stimulated by one research question: *What is the current privacy status of top-ranked mental health apps?* Here, we adopt a broad definition of privacy that encompasses security and data protection and with emphasis on the negative privacy impacts on data subjects.

The methodology for this investigation relied on a range of penetration testing tools and methods for systematic analysis of privacy policies and regulatory compliance artefacts. We selected a sample of 27 top-ranked mental health apps from the Google Play Store that collected, stored and transmitted sensitive personal health information of users. We subjected the apps to static and dynamic security analysis and privacy analysis using various tools. Particular focus was put on using the Mobile Security Framework (MobSF), which provides a wide range of static and dynamic analyzing tools. Other tools such as Drozer, Qualys SSL Labs, WebFX, CLAUDETTE and PrivacyCheck were also employed in this study. Furthermore, we documented the privacy issues that we identified for each app by mapping them to the well-known LINDDUN privacy threat categories (i.e., **L** inkability, **I** dentifiability, **N** on-repudiation, **D** etectability, **D** isclosure of information, **U** nawareness and **N** on-compliance) (Deng et al. 2011).

This study’s findings reveal alarming data privacy problems in the mHealth apps used by millions of users, who are likely to expect data privacy protection built in such apps. Our study’s main findings include:

- Most apps pose linkability, identifiability, and detectability threats. This is a risk as some 3rd-parties can link, re-identify and detect the users’ actions and data. Unawareness is also related to such threats, given that apps do not explain (e.g., in the privacy policy) the risks posed by targeted advertising on people experiencing mental problems and the risk of re-identification and disclosure of mental health conditions (e.g., anxiety, depression).
- Only 3/27 app developers responded to our query regarding Privacy Impact Assessments (PIAs), mentioning that they had performed a PIA on their app, and only two of them had made the PIA reports public. That suggests a high non-compliance rate since mHealth apps tend to pose high-risk to the rights and freedoms of users.
- 24/27 app privacy policies were found to require at least college-level education to understand them. The remaining 3/27 apps needed 10th–12th-grade level education to understand them. Such findings also suggest further problems with regards to non-compliance, leading to data subject’s unawareness about the nature of the data processing activities in mental health apps, data controllers, and service providers.
- Static analysis reports show that 20/27 apps are at critical security risk, and 4/27 apps are at high security risk. Many of the issues are revealed through a simple static analysis, such as the use of weak cryptography. Dynamic analysis also shows that some apps transmit and log personal data in plain-text. Four apps can leak such sensitive data to 3rd-parties, exacerbating risks of (re-)identification and information disclosure.

We have also synthesised the main findings and mapped them according to the LINDDUN privacy threat taxonomy (Deng et al. 2011). The findings highlight the prevalence of data privacy problems among the top-ranked mental health apps. It is clear that companies and software developers should pay more attention to privacy protection mechanisms while developing mHealth apps. At the same time, users and mental health practitioners should demand for (at least) compliance with privacy standards and regulations. Based on the findings, we offer some recommendations for mHealth apps development companies, apps developers, and other stakeholders.

## Background

### Privacy (and Security)

Until quite recently, the term privacy was treated under the umbrella of security. However, this situation has changed with data privacy gaining significance and prominence of its own. It is essential to clarify the difference between privacy and security for the research reported in this paper. In this study, we are mainly interested in data privacy that can be compromised as a result of security breaches. The concept of privacy comprises several aspects such as informed consent, transparency, and compliance, that are not necessarily connected to security. Whilst privacy is protected through security measures, privacy cannot be satisfied solely on the basis of managing security (Brooks et al. 2017). For such reasons, we regard security as part of a broad conceptualisation of privacy, which includes protecting personal data. As a consequence, the study design reflects this contrast between privacy and security. That is, apart from traditional security testing, this study also evaluates the apps’ privacy policies, makes requests for privacy impact assessments, and gathers the developers’ feedback on raised issues.

### The ecosystem of mental health Apps

**Fig. 1:**
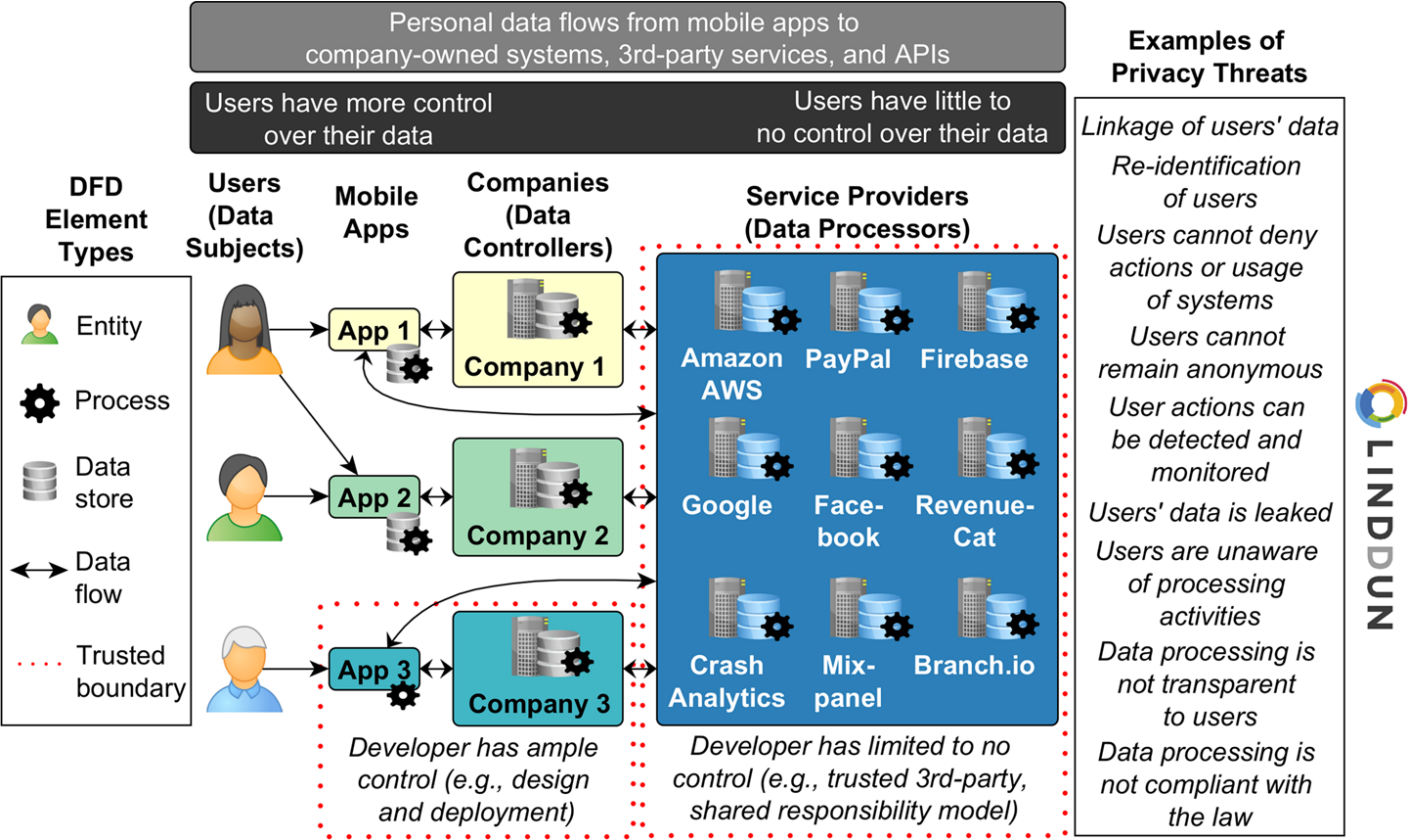
Simplified Data Flow Diagram (DFD) for the apps’ ecosystem with an overview of the data subjects, data controllers, data processors, and privacy threats to consider

Today’s information systems are built upon a wide range of services involving multiple stakeholders. Figure 1 presents a simplified Data Flow Diagram (DFD) that can help a reader to identify the main actors in the mental health apps ecosystem for discussing the privacy issues. As shown in Fig. 1, users (i.e., data subjects) have their data collected by mHealth apps and transmitted to the companies (i.e., data controllers) as well as to the other service providers (i.e., data processors). Privacy considerations should be made for every step of the DFD (i.e., a detailed DFD created by apps developers) in which personal data is processed, stored and transmitted.

First, as shown in Fig. 1, the personal data flows from an app to a company-owned server. Here developers have a greater control on the system’s design so that the main concern is the protection of data at-rest, in-transit and in-use. Developers can fully understand all aspects of the company-owned infrastructure (i.e., client and server sides). Thus, they can transparently communicate the nature of personal data collection and processing to its users. Data flows within this trusted boundary of the company-owned systems tend to be less problematic regarding privacy. However, it is worth stressing that privacy goes beyond data protection, so other privacy aspects should be considered, such as unawareness and non-compliance threat categories.

Second, personal data flows to many 3rd-party service providers that support the collection and processing of the users’ data. Most companies rely (often entirely) on public cloud infrastructures (e.g., Amazon AWS, Google Cloud) to maintain their servers and databases, as well as use many APIs that provide services for the apps to function (e.g., CrashAnalytics, RevenueCat, PayPal, Firebase). In such cases, developers have limited control over the system, and the processing activities are not fully transparent anymore. Developers have to trust service providers, and a shared responsibility model ensues. Thus, the data flows going to service providers should be carefully scrutinized. This concern is particularly critical in the context of mental health apps since the personal data is considered highly sensitive, as previously mentioned.

Adding to the problem, companies often rely on advertising as a source of monetary income for their apps, and mental health apps are no exception in such business models. Thus, a user’s information provided for using an app may be distributed to the app developer(s), to 3rd-party sites used for functionality reasons, and to unidentified 3rd-party marketers and advertisers (Giota and Kleftaras 2014). Whilst users and health professionals are expected to be aware of such risks, it is important that companies that develop mHealth apps are also transparent about the business model in which they operate. Users already have little control over their data that resides within the developers’ systems, let alone the data shared with 3rd-parties, such as mobile advertising platforms and data brokers.

### The LINDDUN threat taxonomy

LINDDUN is a well-known privacy threat modelling framework (Deng et al. 2011), recently included in the NIST Privacy Framework (NIST 2022). Given the increasing popularity of LINDDUN framework for systematically analyzing privacy threats during software systems development, we decided to use LINDDUN to analyze and map the findings from our study. The LINDDUN privacy threat analysis methodology consists of three main steps: (1) modelling the systems, using DFDs and describing all data; (2) eliciting privacy threats, iterating over the DFD elements to identify threats using a taxonomy; and, (3) managing the threats, finding suitable solutions to tackle the uncovered threats.

We are mainly interested in the LINDDUN threat taxonomy, which can be used as a standard reference for discussing privacy threats:

- Linkability: an adversary can link two items of interest (IOI) without knowing the identity of the data subject(s) involved (e.g., service providers are able to link data coming from different apps about the same data subject).
- Identifiability: an adversary can identify a data subject from a set of data subjects through an IOI (e.g., service providers can re-identity a user based on leaked data, metadata, and unique IDs).
- Non-repudiation: the data subject cannot deny a claim, such as having performed an action or sent a request (e.g., data and transactions stored by companies and service providers cannot be deleted, revealing the users’ actions).
- Detectability: an adversary can distinguish whether an IOI about a data subject exists or not, regardless of being able to read the contents itself (e.g., attackers can detect that a user’s device is communicating with mental health services).
- Disclosure of information: an adversary can learn the content of an IOI about a data subject (e.g., data is transmitted in plain-text).
- Unawareness: the data subject is unaware of the collection, processing, storage, or sharing activities (and corresponding purposes) of the data subject’s data (e.g., the companies’ privacy policy is not easy to understand and transparent about the nature of data processing).
- Non-compliance: the processing, storage, or handling of personal data is not compliant with legislation, regulation, and policy (e.g., a company fails to perform a PIA for a privacy-sensitive systems).

Each of these seven threat categories is composed by distinct threat trees, forming the complete threat taxonomy. For instance, the Linkability category is subdivided into four threat trees: (1) Linkability of Entity (L_e); (2) Linkability of Data Flows (L_df); (3) Linkability of Data Store (L_ds); and, Linkability of Process (L_p). Each of the threat trees is modeled in a number of branches in which the leaf nodes refer to a specific threat. For instance, if we take the threat tree of Linkability of Data Flow (L_df), it develops in two main branches, i.e., Linkability of transactional data (transmitted data) (L_df1) and Linkability of contextual data (metadata) (L_df2). These two branches are then divided into other more specific threats, e.g., data flow not fully protected (L_df6) or linkability based on IP address (L_df8). The other threat trees, i.e., Linkability of Entity and Linkability of Data Store, follow the same overall structure of branches and leaf nodes.

However, not all of the main threat categories are composed of multiple threat trees. The category of Unawareness, for example, contains only the threat tree for Unawareness of Entity (U_e); this is the only relevant, i.e., only an entity can be unaware, not a data flow, data store, or process. And particularly for the threat tree of Information Disclosure, the LINDDUN methodology actually borrows its threat trees from Microsoft’s security threat model, STRIDE (Howard and Lipner 2006).

For a complete account of all the LINDDUN threat categories, threat trees, and specific threats, we refer the reader of this article to the catalogue compiled in Wuyts et al. (2014). Some familiarity with LINDDUN is beneficial since we refer to specific threats throughout the paper, e.g., when describing how LINDDUN was incorporated into our research methodology for this study and when discussing the main findings and results.

### Related work

#### Security and privacy of mHealth Apps in general

The broad category of mHealth apps includes several types of apps, such as wellness and fitness apps (e.g., calorie counters, exercise trackers), personal health apps (e.g., diabetes monitors, symptom checkers), and medical resource apps (e.g., drugs catalogues, medical terminology libraries). In the past years, many studies have analyzed the security and privacy of mHealth apps in general. Some studies focus on the analysis of the more “visible” aspects of the mHealth apps, looking into their privacy policies, user interfaces, documentation, and websites (Adhikari et al. 2014; Sampat and Prabhakar 2017; Hutton et al. 2018; Shipp and Blasco 2020).

For instance, the work of Hutton et al. (2018) contributed with a set of heuristics for evaluating privacy characteristics of self-tracking apps. They introduced 26 heuristics under the four categories of (a) notice and awareness, (b) choice and consent, (c) access and participation, (d) social disclosure usability. A group of 4 HCI and software engineering researchers then analyzed 64 mHealth apps for self-tracking using these heuristics by reviewing the apps’ user interface, terms of service, and privacy policies, reaching a moderate agreement (*kappa* = .45) (Hutton et al. 2018). This work mentions that disagreements between raters mainly arose from confusion over the privacy policies that are often unclear regarding language and intent. We can also add that privacy lawyers would be better suited for this type of analysis because the apps’ terms of service and privacy policies are legal artefacts. Nonetheless, their results show that most apps performed poorly against the proposed heuristics, the app maturity was not a predictor for enhanced privacy; and apps that collected health data (e.g., exercise and weight) performed worse than other self-tracking apps (Hutton et al. 2018). Adhikari et al. (2014) and Sampat and Prabhakar (2017) have also warned about the issues concerning insufficient privacy policies (e.g., unclear or non-existent), lack of data access and deletion functions, and opaqueness in the data sharing with 3rd-parties. Shipp and Blasco (2020) also looked into menstrual apps in order to show that developers often fail to consider menstruation and sex data as especially sensitive, mentioning only common pieces of personal data (e.g., name, email) in their privacy policies.

Other studies have privileged the “invisible” aspects of mHealth apps’ security and privacy, e.g., using pentesting tools to analyze the apps’ code, network traffic, logs, and generated data (He et al. 2014; Papageorgiou et al. 2018; Hussain et al. 2018; LaMalva and Schmeelk 2020). The earlier work of He et al. (2014) expressed concerns about the widespread use of unsecured Internet communication and 3rd-party servers by mHealth apps. Papageorgiou et al. (2018) carried out a more in-depth security and privacy analysis, revealing several vulnerabilities, such as unnecessary permissions, use of insecure cryptography, hard-coding and logging of sensitive information, insecure server’s SSL configuration, and transmission of personal data to 3rd-parties. Similar threats have also been identified in other studies as reported by Hussain et al. (2018) and LaMalva and Schmeelk (2020).

The above-mentioned studies have contributed significantly to the researchers’ and practitioners’ understanding of security and privacy threats in mHealth apps in general. However, these studies often analyze mHealth apps in wellness and fitness categories instead of apps with highly sensitive data such as those in the mental health area. From 2020 to 2022, a sharp increase of users have turned to mental health apps as an effect of the COVID-19 pandemic; this context motivated our research team to perform this study. Nonetheless, even after the pandemic, this trend will continue with the increased adoption of mental health services supported by mHealth technologies.

#### Security and privacy of mental health Apps

As shown in Table 1, we identified eight studies related to the security and privacy of mental health apps. However, the existing related work has a limited scope of analysis. Most researchers focus only on the apps’ privacy policies (O’Loughlin et al. 2019; Powell et al. 2018; Robillard et al. 2019; Rosenfeld et al. 2017). Another work investigates only the apps’ permissions (Huang and Bashir 2017), or the combination of apps’ permissions and privacy policies (Parker et al. 2019). Another study (Muchagata and Ferreira 2019) proposes a scope of analysis to check for GDPR compliance, i.e., assessing the types of collected data, apps’ permissions, and evidence of consent management, data portability and data deletion features. Such approaches mostly reveal Unawareness and Non-compliance issues, missing the other categories of privacy threats. That means their results do not have the depth of penetration tests to identify the presence of the concrete privacy threats.

**Table 1:** Comparison of the existing works on privacy and/or security for mental health apps according to their scope of analysis

Ref	Year	N. of Apps	Condition	PP	PIA	Per	DT	Code	Log	DS	SC	UC	Limitations
Huang and Bashir (2017)	2017	274	Anxiety			✓							(i) Limited to anxiety apps. (ii) Analyzes only apps’ permissions.
Huckvale et al. (2019)	2019	36	Depression and smoking cessation	✓			✓						(i) Limited to depression and smoking cessation apps. (ii) Analyzes only the privacy policies and network traffic.
Muchagata and Ferreira (2019)	2019	18	Dementia	✓		✓						✓	(i) Limited to dementia apps. (ii) Analyzes only PPs and permissions and performs a GDPR compliance check.
O’Loughlin et al. (2019)	2019	116	Depression	✓									(i) Limited to depression apps. (ii) Analyzes only the PPs.
Parker et al. (2019)	2019	61	Mental health	✓		✓							(i) Analyzes only the apps’ permissions and PPs.
Powell et al. (2018)	2019	70	Diabetes and mental health	✓									(i) Analyzes only the complexity of PPs.
Robillard et al. (2019)	2019	369	Track and mood	✓									(i) Analyzes only the PPs and terms of agreement.
Rosenfeld et al. (2017)	2017	33	Dementia	✓									(i) Limited to dementia apps. (ii) Analyzes only the PPs.
This work		27	Mental health	✓	✓	✓	✓	✓	✓	✓	✓	✓	(i) Limited to top-ranked mental health apps. (ii) PPs analyzed using AI-assisted tools.

**Abbreviations:** Privacy Policy (PP), Privacy Impact Assessment (PIA), Permissions (Per), Data Transfer (DT), Data Stored (DS), Server Configuration (SC), User Control (UC)

One study has also examined the apps’ network traffic and data transmissions, in addition to assessing the privacy policies (Huckvale et al. 2019). Looking into the network traffic enabled the identification of data that is transmitted to 3rd-parties, such as marketing and advertising services. To some extent, this study may cover all LINDDUN threat categories, but it misses many branches in the LINDDUN threat trees. For instance, logs and stored data are not inspected for data leaks and weak access control; nor is the reverse engineered code reviewed for insecure coding. These types of inspections are important in order to achieve breadth and depth of privacy analysis.

In this work, we employed an extensive assessment framework for the privacy analysis of mental health apps, detailed in Section 3. In brief, our privacy analysis work included a series of penetration tests, with static and dynamic analysis, inspecting apps’ permissions, network traffic, identified servers, reverse-engineered code, databases and generated data, which had not been explored in the related work shown in Table 1. Furthermore, the proposed privacy analysis also involves communication with companies and software developers by requesting the PIAs of the apps and discussing findings through the responsible disclosure process.

## Methodology

**Fig. 2:**
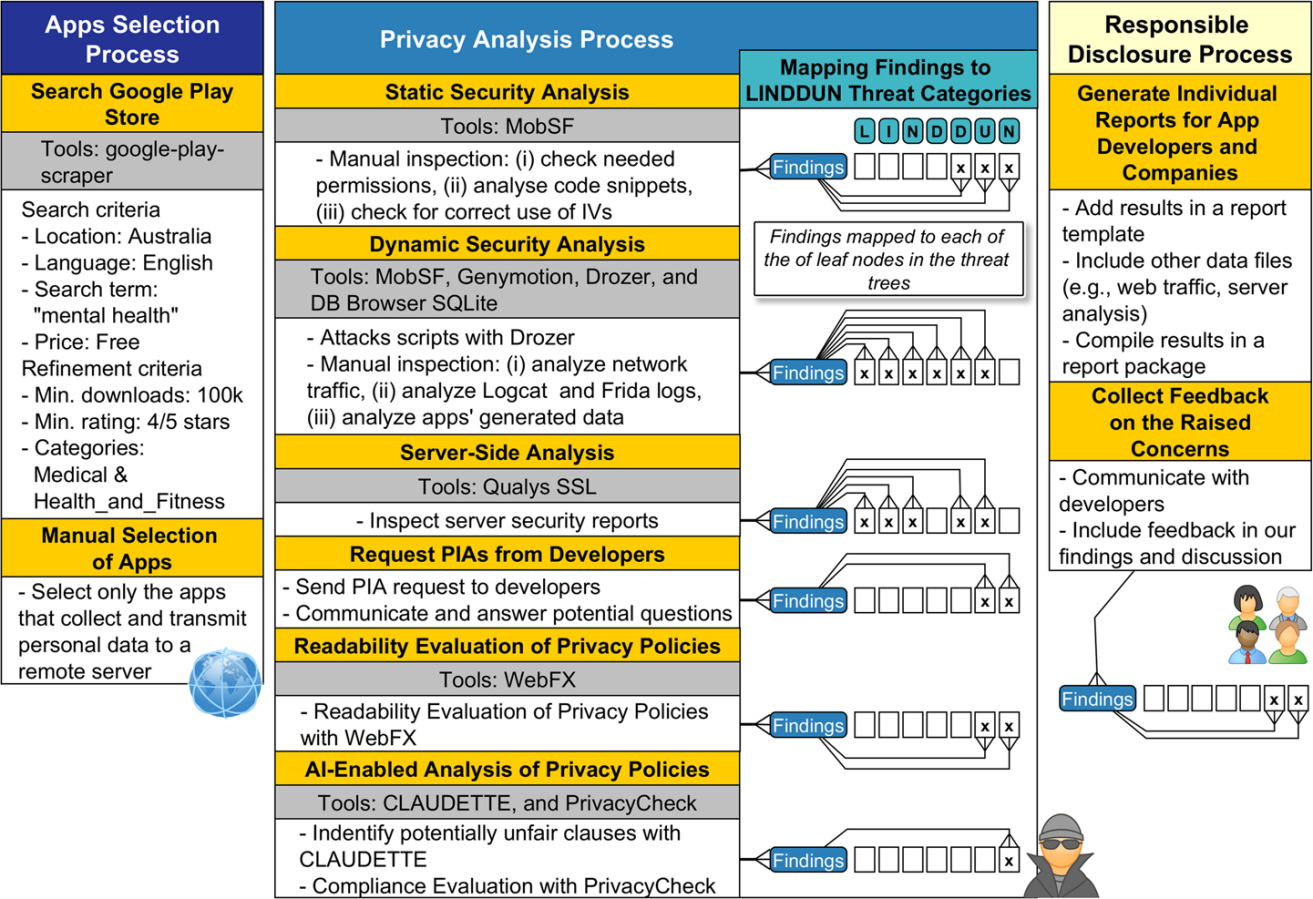
An overview of the methodology used for investigating the privacy issues in mental health apps

This section presents the methodology used for the privacy assessment of the mental health apps. Figure 2 gives an overview of the main processes, specific steps, and tools used throughout the study.

### Apps selection process

For this study, we selected mobile applications developed for Android devices that can be downloaded from Google Play Store. The initial identification of potential apps for the study was performed using the google-play-scraper Node.js module^1^, essentially searching for free mental health apps in English (see Fig. 2), and setting the location to Australia.

This search resulted in 250 apps as 250 is the default maximum number set by Google Play Store. In this study, we are particularly interested in top-ranked mental health apps. The main reason for focusing on top-ranked apps is that we sought to concentrate efforts on the most popular mental health apps, in which privacy impacts may affect millions of users. In order to select only the top-ranked apps, we introduced the following refinement criteria during the app selection process: apps should have at least 100K downloads, rating above 4 stars, and categorized as MEDICAL or HEALTH_AND_FITNESS. This refinement reduced our sample to 37 Android apps.

We also wanted to limit our analysis to apps that require health and/or personal data as inputs in order to be functional and transmit users’ data to a remote host. That is, to avoid analyzing apps data do not collect any personal data, e.g., a mindfulness app that only plays music would most likely have fewer privacy impacts. To identify these types of apps, we manually inspected the apps to figure out whether they store and transmit personal data of their users. This process was carried by two researchers that jointly tested the apps and reached a consensus on whether to include or exclude the app from the study. There were no disagreements between the researchers in this step. This manual analysis included several tasks such as downloading the apps, reading their descriptions, creating and using dummy accounts to use the apps, entering information and checking apps’ functionalities. The analysis stopped once we gathered sufficient evidence. That is, if an app collects and stores or transmits at least one item of personal data (e.g., username, password, email, mood levels, journal entry), we would consider this app for further analysis. We adopted this low threshold for personal data collection because we assumed that even if an app adopted stringent data minimisation strategies, there would still be potential privacy risks given the rather sensitive context of the mental health apps.

This analysis identified nine apps that do not collect and transmit personal/health data of users. Also, one of the apps provided a forum and chatting functionalities to users (e.g., to discuss problems that they face or create support groups). The analysis of this app would reveal information about other users on the platform. The mere collection of personal data of other users (i.e., usernames, posts, replies) would require a complete ethical application to address potential privacy issues. Therefore, we omitted these 10 apps from our analysis and selected the remaining 27 apps to perform the privacy-centred security analysis.

### Privacy analysis process

As shown in Fig. 2, after filtering the 27 apps to perform the analysis, we performed static and dynamic security analysis to identify security vulnerabilities of the shortlisted mental health apps. We also used Qualys SSL to evaluate all the servers identified during the dynamic security analysis. Altogether, these three initial steps of the Privacy Analysis Process are mostly focused on the threats related to linkability, identifiability, non-repudiation, detectatbility and disclosure of information. However, unawareness and non-compliance threats are also detected here but to a lesser extent, e.g., when analyzing apps’ permissions and manifest files.

In parallel, we also sent emails to all developers, companies or DPOs requesting the PIA reports of the studied apps. A readability analysis of the apps’ privacy policies was conducted, and the apps were analyzed using AI-enabled tools to identify unfair clauses and points of non-compliance. Hence, these remaining steps mainly targeted threat categories of unawareness and non-compliance. All steps of the Privacy Analysis Process are detailed in the next sub-sections.

During the study, the researchers used two mobile phones while selecting the apps, an OnePlus 3 (Snapdragon 820, 6GB RAM) and a Moto G4 (Snapdragon 617, 6GB RAM). We also used two laptops: HP Pavilion 15t (Core i7, 16GB RAM) and a Asus X555U (Core i7, 8GB RAM), with Windows 10 as operating system.

#### Static security analysis

We performed the static analysis using an open-source analysis tool called MobSF, which is known for providing a convenient and easy to use fully automated framework for pen-testing 2. MobSF is widely used by security researchers for performing security analysis of mobile applications (Papageorgiou et al. 2018). Furthermore, previous research has shown MobSF’s capability for identifying a wide range of Android security issues (Ranganath and Mitra 2020).

To perform the static analysis, we downloaded the APK file for each app and analyzed it using the MobSF static analyzer. This analysis reveals various details about each app, e.g., including the apps’ average Common Vulnerability Scoring System (CVSS) Score (FIRSTOrg 2019), trackers, certificates, android permissions, hard-coded secrets, and URLs etc.

One of the limitations of this type of analysis is that MobSF may report a considerable amount of false positives related to some vulnerabilities (Papageorgiou et al. 2018). Therefore, based on the initial results obtained by MobSF, we further performed the following checks to verify the issues reported by MobSF.

- Manually evaluate whether or not a “dangerous” permission is required to serve the app’s purpose. For that, MobSF looks into the protection level of a permission that may be specified as dangerous^3^.
- Manually analyze the code snippets that were reported to use insecure random number generators, insecure ciphers and insecure cipher modes.
- Manually checked the code snippets that used IvParameterSpec to test whether Initializing Vectors (IVs) have been correctly used.

The manual analysis process was performed by two researchers that continuously reviewed, discussed and documented the findings for each app. This analysis is based on expert knowledge, i.e., two privacy and security experts with experience in secure programming, mobile security, and mobile and web development. Both researchers jointly read and discussed the static analysis reports generated by MobSF that flagged potential vulnerabilities. Based on our assessment, the relevant issues were then compiled in a separate document listing all the problems for a given app. This manual process occurred in multiple iterations, repeatedly reading and analyzing the data, discussing potential vulnerabilities, and deciding whether or not to mark it as a real issue. The same approach was also used during the dynamic analysis to inspect the apps’ logs, network traffic, generated data and databases, further discussed as follows.

#### Dynamic security analysis

As the next step, we performed the dynamic analysis of the apps. Dynamic Analysis is a black-box security testing methodology that analyzes an app by running it and performing potentially malicious operations on it. For performing dynamic analysis, we used Genymotion Android emulator^4^, MobSF dynamic analyzer and Drozer^5^. The rationale for using MobSF and Drozer is that these frameworks are among the most popular and widely used dynamic analyzers in the field. While MobSF and Genymotion enabled us to emulate the apps, run a series of security checks, and collect various data (e.g., logs, network traffic, databases), Drozer simulated a rogue application that runs attack scripts against a target app. Only 19 apps were subjected to the dynamic analysis in this step as the other eight apps were not compatible to run on the Android emulator with MobSF. One common cause for this incompatibility is that the app is not allowed to run in a rooted device, e.g., the developer uses code to block the app from running in emulation environments. Another cause is that Genymotion’s virtual machines have a x86 (32 bits) architecture and the app is provided for ARM only. We consider this as a limitation of the used methodology.

In the analysis process, we installed each of the studied apps into Genymotion emulator and manually performed various operations on each app while MobSF dynamic analyzer was listening to the performed operations. The manually performed operations consisted of opening and navigating to all pages (i.e., activities) of the apps, inputting text and recording entries with the apps, storing and sending data. Manual operations were performed until we used all the available features in the app, except for the paid ones, which should be considered as a limitation of this study. At the end of the analysis, MobSF provided us with a report that included the complete Logcat log, Dumpsys log, Frida API monitor log and HTTP/S traffic log for the whole period that we were interacting with each app. Furthermore, MobSF allowed us to download all the data created by each app that persisted in the device’s storage. It is worth mentioning that at this step, we were conscientiously exploring the app’s functionalities as opposed to performing other stress and reliability tests (e.g., using random inputs to analyze the app behaviour, crashes, and performance in general), which are not explicitly geared towards revealing security and privacy issues.

In addition, when an app was running on the Android emulator, we used Drozer to perform malicious operations on the app to identify app’s security vulnerabilities (MWR InfoSecurity 2015). We used various attack scripts such as checking for attack surfaces, SQL injection, and directory traversal vulnerabilities.

Thereafter, we performed a detailed analysis of the logs and apps’ data files that were obtained from MobSF dynamic analysis. The HTTP/S traffic log provided the request and response information for each HTTP/S communication made during the process. We went through the log entries for each communication and investigated for any insecure channels that might have communicated users’ sensitive data (e.g., health data, location, email, password). Furthermore, we also checked for the 3rd-party servers that each app was sending users’ personal and health data.

In the next step, we analyzed Logcat logs and Frida API monitor logs generated during the dynamic analysis process to identify whether or not these logs reveal personal information of a user, reveal apps’ behavior, usage, and activities, reveal tokens and credentials used by the app, or reveal the details of web traffic, parameters and Post values. Logcat logs generated in the device are accessible to other apps running on the device and logging sensitive information make such information accessible to those apps (Kotipalli and Imran 2016). To identify these insecure log entries, we first searched in the log files for various obvious keywords of which the useful ones included: ‘username’, ‘password’, ‘API key’, ‘api-key’, ‘api_key’, ‘key’, ‘@gmail.com’, ‘mood’, ‘meditat*’, ‘checkup’, ‘assessment’, ‘login’, ‘http*’, ‘post’, ‘uuid’, ‘aaid’, ‘save’, ‘delete’. Two of the researchers involved in this study subsequently read all the log files looking for other suspicious entries.

As the final step of the dynamic analysis process, we analyzed the data generated by each app (i.e., files and databases) to see whether or not an app had insecurely stored any user’s sensitive data. We categorized the data as encrypted or not encrypted, and used DB Browser SQLite^6^ to open and browse all the data stored in the apps’ folders, files and databases. Similar to the other manual analysis processes, we read all files looking for sensitive data that had been stored insecurely.

#### Server-side analysis

We also performed web server analysis on each domain with which the app communicated during the dynamic analysis. As part of this step, the relevant web servers’ configurations were analyzed to assess the security levels of the HTTPS data transmissions. To perform this analysis, we used Qualys SSL Labs^7^ tool, which is a free online service that enables the remote testing of web server’s security against a number of well-known vulnerabilities, such as Heartbleed (Durumeric et al. 2014) and Drown (Aviram et al. 2016). The analysis provided an overall rating for the web server’s security (A+, A, B, C, D, E, F) as well as a score and weaknesses for certificate, protocol support, key exchange and cipher strength aspects.

#### Request privacy impact assessment

Privacy Impact Assessment (PIA), also known as Data Protection Impact Assessment, is an important component of an app’s accountability that comes under GDPR (ICO UK 2019). Most information privacy regulations, such as the GDPR and the Australian Privacy Act, encourage the publication of PIA reports as it demonstrates to stakeholders and the community that the project has undergone critical privacy analysis, potentially reducing community concerns about privacy (GDPREU 2020; OAIC 2020). Therefore, as a part of our privacy analysis, we evaluated whether or not the developers of the studied apps had performed PIA on their respective apps and made the findings public. We contacted the companies and/or developers of the studied apps based on the contact details available on Google Play Store and requested them to send the details of the public reports of their PIAs.

#### Readability evaluation of privacy policies

Privacy policies are responsible for communicating how an app gathers, uses, discloses, and manages the personal information of the app users (Zaeem and Barber 2020). Previous research has evaluated privacy policies of different types of apps and reported that privacy policies are often too complex and difficult for users to read and understand (O’Loughlin et al. 2019; Powell et al. 2018). We were interested in evaluating the readability of privacy policies of the mental health app as these apps are often used by users who are already psychologically and cognitively challenged (Marvel and Paradiso 2004).

Therefore, as a part of the privacy analysis step, we evaluated the readability of the apps’ privacy policies. We used WebFX^8^ free online tool for this. This tool provides various readability scores (e.g., Flesch-Kincaid, Gunning Fog, SMOG), as well as a number of metrics about the privacy policies (e.g., number of words, sentences, complex words).

#### AI-enabled analysis of privacy policies

We performed the final component of the privacy policy analysis using two AI-enabled tools, which are CLAUDETTE (Lippi et al. 2019) and PrivacyCheck (Zaeem and Barber 2020; Zaeem et al. 2018). First, we used CLAUDETTE to identify the potentially unfair clauses in apps’ privacy policies, e.g., jurisdiction disputes, choice of law, unilateral termination or change. In addition, we used PrivacyCheck, which is an automated tool provided as a Chrome browser plugin. It evaluates the privacy policy of an app with respect to 20 points criteria where 10 questions are related to users’ control over their privacy and 10 questions are related to GDPR.

### Responsible disclosure process

After completing the Privacy Analysis Process of the selected apps, we prepared the reports on the results for each app. We emailed the evaluation reports to the companies and/or developers of the apps based on the contact details available on Google Play Store and asked them to respond within 30 days whether or not they had fixed the identified security and privacy issues. We gathered the information about how they responded to our report and whether or not they improved their apps based on our findings.

### Mapping findings to LINDDUN

As the last step, a detailed mapping exercise was performed. Essentially, throughout the privacy analysis, a list of privacy issues was compiled for each app. Then, we followed the knowledge support provided by the LINDDUN methodology for cross-checking every single issue in the list with respect to the entire threat taxonomy (threat-by-threat) to check for correspondence. Finally, if one of the threats is relevant to a given issue, this threat is mapped and included in the mapping table (readers are referred to the LINDDUN’s threat tree catalog (v2.0) (Wuyts et al. 2014) for consultation).

**Fig. 3:**
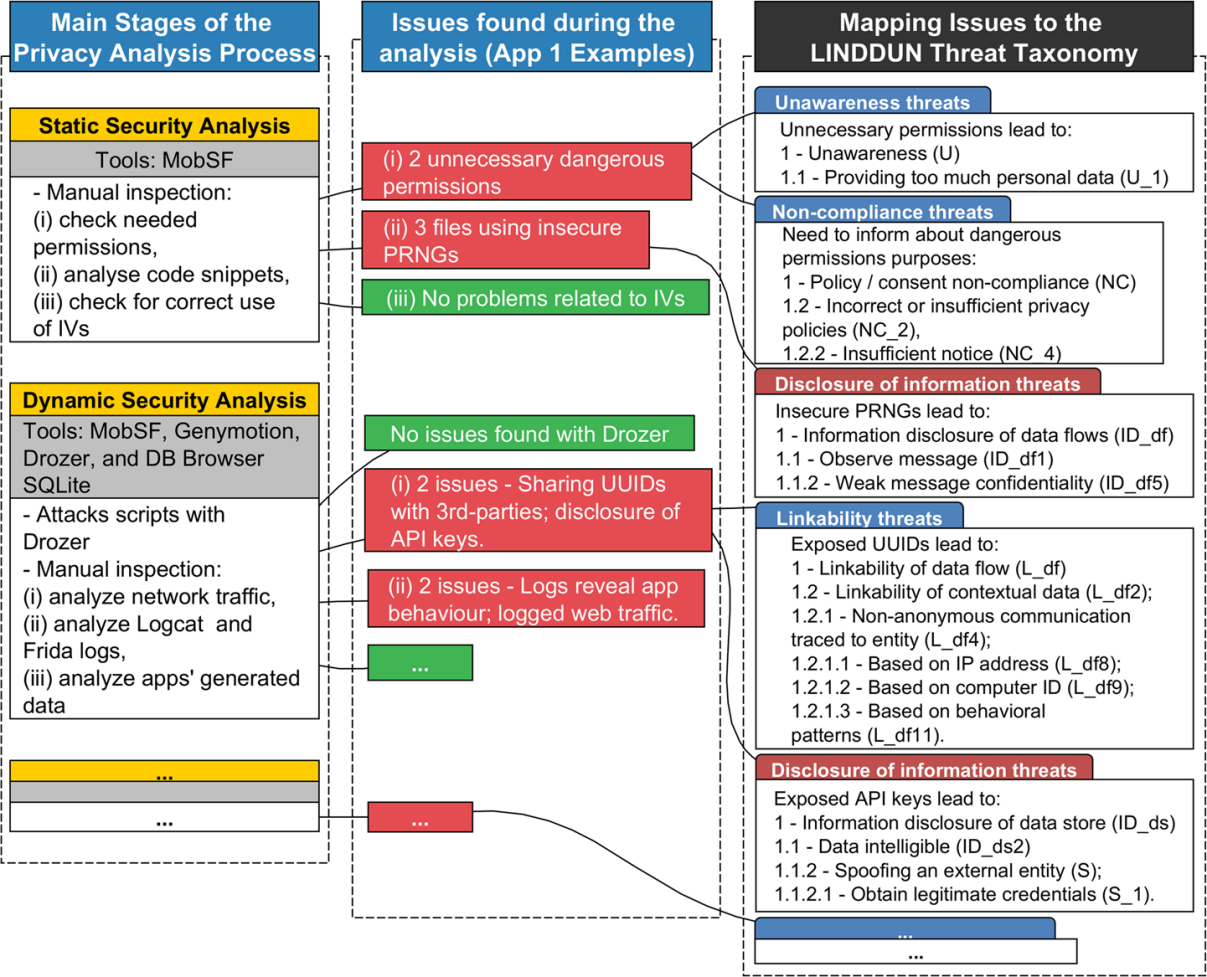
Example of mapping process for App 1: associating issues found during the analysis to the LINDDUN taxonomy threats

An illustrative example is provided in Fig. 3. Every step of the Privacy Analysis Process allows identifying a number of issues. For instance, during the Security Static Analysis of App 1, two dangerous permissions were identified, and three files in the reversed engineered code used insecure PRNGs. One dangerous permission is the android.permission.READ_PROFILE, which allows the application to read the user’s profile data. This permission does not seem necessary at installation time nor for the app to function for its specified purposes, thus it was marked as an issue. Having such dangerous permission results in providing too much data (i.e., Unawareness threat). Also, it relates to insufficient notice to users (i.e., Non-compliance threat tree) since the privacy policy could have better explained the need for this dangerous permission. Similarly, the issues regarding the use of insecure PRNGs may lead to insecure security implementations, and thus, weak message confidentiality (i.e., Disclosure of Information threat). These overarching findings are presented in Section 4 along with the results.

Another way to consider the mapping in Fig. 3 refers to the associations between the main stages of the Privacy Analysis Process (and its respective tools) and the LINDDUN threat taxonomy. For instance, the Static Security Analysis using MobSF allows for identifying “dangerous” permissions and inspection of reverse-engineered code of the apps. As mentioned, the analysis of permissions usually translates into Unawareness and Non-compliance threats. However, it can also lead to Disclosure of Information threats, e.g., if the app uses the permission WRITE_EXTERNAL_STORAGE it might leak information to other apps in the device that can also access the external storage. Similarly, when analyzing the reverse-engineered code for insecure implementations, the threats are mainly associated with the Disclosure of Information since improper cryptography weakens an app’s security, leading to confidentiality breaches.

The Dynamic Security Analysis was also crucial for gathering various data generated by the apps, especially in terms of data flows (e.g., network traffic and system logs) and data stored (e.g., files and databases). Privacy issues found in data flows are associated with several threats in terms of Linkability, Identifiability, Non-repudiation, Detectability, Disclosure of Information, and Unawareness. For instance, data flows can be linked based on unique IDs, some data points (e.g., IMEI, location) can facilitate the re-identification of individuals, logging and data sharing thwart plausible deniability, data flows can be easily detected (revealing the usage of an app), personal data might leak to 3rd-parties (e.g. email address, username), and finally, users can be unaware about such extensive profiling and data sharing operations. Arguably, the dynamic analysis of apps is essential to identify and verify privacy issues related to many of the LINDDUN’s threat categories.

The other stages of the Privacy Analysis Process can also be mapped onto LINDDUN taxonomy. The Server-Side Analysis focuses on the server’s security configuration. Thus, it mainly relates to the Disclosure of Information threats, such as using weaker versions of communication protocols. The stages such as Request of PIAs, Readability of Privacy Policies, and AI-Enabled Analysis of Privacy Policies reveal problems in terms of Unawareness and Non-compliance, e.g., the nature of data collection and processing can be hard to understand, or it misses relevant information, or unfair clauses are used. Lastly, further communication in the Responsible Disclosure process can also be associated with LINDDUN taxonomy, such as failing to provide relevant information and ignoring the data subject’s requests.

## Results

### Selected mental health Apps

The final sample consists of 27 Android apps that provide functionalities related to mental health services. Twenty-one of the selected apps were from the ‘Health & Fitness’ genre; the remaining six apps were from the ‘Medical’ genre. Table 2 provides a summary of the sample of apps used in this study. The selected apps originate from 11 different countries from four continents. To keep the apps de-identified, we have not included the exact details about the apps’ origin countries.

**Table 2:** Apps with their respective number of downloads

N. of Apps	N. of Downloads
12	100,000 - 500,000
3	500,000 - 1,000,000
9	1,000,000 - 5,000,000
1	5,000,000 - 10,000,000
2	10,000,000+

Table 3 provides the results of a tagging exercise performed by the researchers for all the selected apps. We read the apps’ contents from Google Play Store and created tags about the app’s main scope in terms of mental health (e.g., stress, anxiety, depression), functionalities (e.g., journal, trackers, diagnosis), and other relevant tags (e.g., online therapy, peer-support). Our approach follows the method of *“generating initial codes”* (Braun and Clarke 2006), in which the codes/tags are mostly descriptive and based on explicit terms and words used in the apps’ contents. A total of 36 tags were generated. Each app was tagged with two to nine tags representing their scope, which allowed us to group them into themes. Notice that an apps may fall into one or more themes. As shown in Table 3, Anxiety, Stress and Depression are the most common tags among the selected apps. This tagging exercise provides an overview of the apps’ themes while keeping the apps de-identified.

**Table 3:** Themes of analyzed apps

N. of Apps	Tags
22	Anxiety
19	Stress and burnout
13	Depression
13	Sleep and insomnia
13	Journal, diary and daily-planner
12	Mood and habit tracker
10	Disorders, addiction, bipolar, anger, phobia, eating disorder, negative emotions, mood disorder, self-harm, PTSD, OCD, and ADHD
8	Meditation
8	Panic attack
8	Online therapy, online doctor, and couples therapy
5	Chatbot
5	Other, e.g., peer-support, pregnancy, pain management, bullying
4	Self-esteem
3	Mental health assessment, diagnosis, and check symptoms

Of the 27 top-ranked mental health apps selected, most address the conditions of anxiety, stress, burnout and depression. Also, over a third of them address various other mental health conditions, e.g., addictions, bipolar, self-harm, PTSD and OCD. For these reasons, we argue that these apps’ processing operations ought to be considered “high-risk” to the rights and freedoms of their users.

### Summary of results according to LINDDUN

This section summarises the mapping between the identified issues for a given app and the LINDDUN threat categories – this data is provided as supplementary material in the file “Mapping Apps’ Issues to LINDDUN”. As shown in Table 4, considering App 1, three of the found issues were mapped to one or more of the Linkability threats. On average, we observed that apps had 22.3 privacy issues, ranging from 1.8 to 6.0 for each individual category. In what follows, we structure the results section around the seven LINDDUN threat categories, covering the main threats that manifest in the studied apps. Evidence gathered during the Privacy Analysis Process, such as the results of tools (e.g., MobSF, Qualys SSL, CLAUDETTE) and manual analysis of network traffic and logs, are used as examples of how the threats appear.

**Table 4:** Mapping summary, showing the number of times that one of the apps’ issues was mapped to a threat category. Note: (*) means that the dynamic analysis could not be performed for the app

App code	L	I	N	D	D	U	N	Total
App 1	3	3	3	3	5	5	7	29
App 2	2	2	2	2	6	4	5	23
App 3	2	2	2	2	7	4	6	25
App 4*	–	–	–	–	4	4	6	14
App 5	2	2	2	2	7	4	5	24
App 6*	–	–	–	–	2	4	5	11
App 7	3	3	3	3	6	5	7	30
App 8*	–	–	–	–	3	4	6	13
App 9	2	2	2	2	6	4	6	24
App 10	2	2	2	2	6	4	6	24
App 11	3	3	3	3	7	4	5	28
App 12	3	3	3	3	6	5	7	30
App 13	3	3	3	3	6	5	7	30
App 14	3	3	3	2	5	5	7	28
App 15	3	3	3	3	6	5	7	30
App 16	3	3	3	3	7	5	7	31
App 17	3	3	3	3	6	5	7	30
App 18	3	3	3	3	7	5	6	30
App 19*	–	–	–	–	2	4	6	12
App 20	2	2	2	2	4	3	4	19
App 21*	–	–	–	–	1	4	6	11
App 22*	–	–	–	–	1	4	6	11
App 23	3	3	3	3	7	5	7	31
App 24*	–	–	–	–	1	4	6	11
App 25*	–	–	–	–	2	4	6	12
App 26	2	2	2	2	4	3	4	19
App 27	2	2	2	2	5	4	6	23
**Avgs.:**	1.8	1.8	1.8	1.8	4.8	4.3	6.0	22.3

#### Linkability threats

LINDDUN borrows most of its terminology definitions from the work of Pfitzmann and Hansen (2010), including the definition for linkability. Linkability is the ability to sufficiently distinguish whether two IOI are linked or not, even without knowing the actual identity of the subject of the linkable IOI. Typical examples are anonymous letters written by the same person, web pages visited by the same user, entries in two databases related to the same person, people related by a friendship link, etc.

Such linkability threats are revealed during the dynamic analysis of an apps when network traffic was manually inspected. This is done using tools such as MobSF and Genymotion to emulate apps and capture their network traffic, logs, and generated data.

**Fig. 4:**
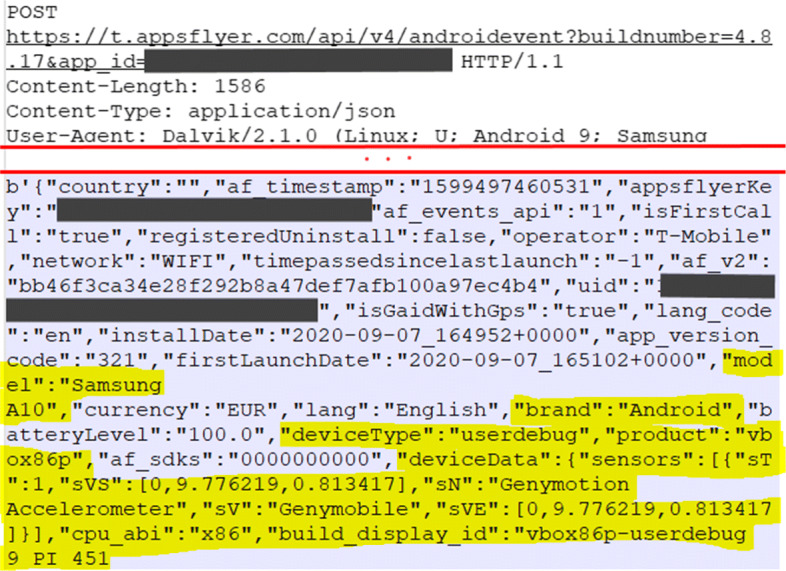
Example of 3rd-party receiving user’s device information

The most prevalent type of threat refers to the linkability of contextual data (L_df2) concerning data flows. Contextual data becomes linkable when non-anonymous communication (L_df4) is used, which is the reality for all the selected apps. Hence, the data flow can be linked based on IP address, device IDs, sessions IDs, or even communication patterns (e.g., frequency, location, browser settings). An example of an app sharing linkable data to 3rd-parties, such as the user’s usage and activities in the app or the device configuration, is shown in Fig. 4. In this case, linkable data is shared with AppsFlyer, a mobile marketing analytics and attribution platform.

**Fig. 5:**
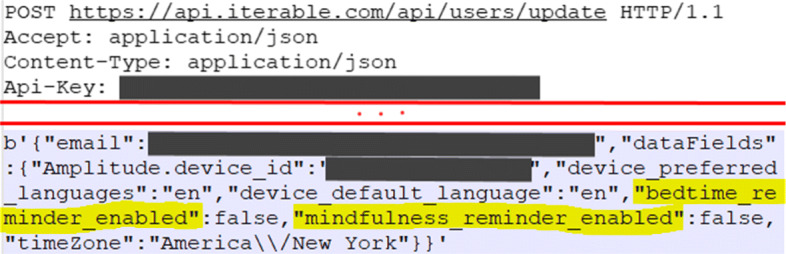
Example of 3rd-party receiving user’s activities information

Such linkability threats manifested in all the 18 apps that went through the dynamic analysis. User behaviour can be easily extracted from web traffic logs (i.e., it is easy to perform profiling of mental health apps’ users), even if one cannot re-identify a subject (see Fig. 5). Most apps also attempt to pseudo-anonymize users through anonymous IDs or hashed advertisement IDs, but these IDs can still be used to link data among various 3rd-parties. In particular circumstances, two apps exacerbate linkability threats by generating a perplexing number of HTTP(S) requests in a short period (i.e., App 23 made 507 and App 15 made 1124 requests). The more data is available, the worst it is in terms of linkability. More data points are linked over longer periods of time; and it is also harder to hide the links between two or more items of interest (e.g., actions, identifiers).

#### Identifiability threats

Identifiability of a subject from an attacker’s perspective means that an attacker can sufficiently identify a subject within a set of subjects (Pfitzmann and Hansen 2010). Examples are identifying the reader of a web page, the sender of an email, the person to whom an entry in a database relates, etc. It is worth mentioning that likability threats increase the risks of re-identification. The more information is linked, the higher the chance the combined data are identifiable (i.e., the more attributes are known, the smaller the anonymity set).

**Fig. 6:**
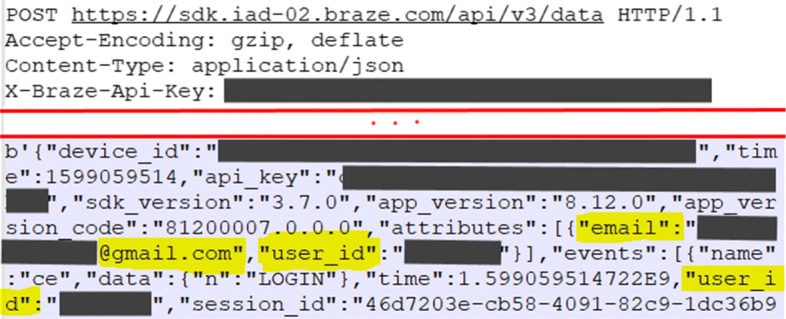
Example of 3rd-party receiving user’s email information

Identifiability threats are also revealed through the dynamic analysis when inspecting network traffic, logged and stored data, using tools such as MobSF, Genymotion, Logcat dumps, and DB Browser SQLite. Here we are particularly interested in data flows that go to 3rd-parties or that may be accessible by attackers (i.e., situations in which users typically assume that they are anonymous). Identifiability of log-in used (I_e1) and contextual data (I_df2) were the most common types of threat found in the 18 apps that went through dynamic analysis. In such cases, users can be re-identified by leaked pseudo-identifiers, such as usernames and email addresses, as shown in Fig. 6.

Identifiability may also manifest due to weak access control to stored data (I_ds1). These situations were observed when apps leak personal information in the system logs (accessible by all apps), or store data in plain text, using databases or external storage. However, attackers would need physical access to the device to exploit such threats, and in such cases, it is likely that they already know the victim’s identity. Such types of threats are nonetheless discussed under the threat category of Disclosure of Information in Section 4.2.5.

#### Non-repudiation threats

Non-repudiation refers to not being able to deny a claim or action. Therefore, an attacker can prove that a user knows, has done, or said something, such as using mental health apps and services. Here, again, we are particularly interested in non-repudiation threats involving 3rd-party systems.

**Fig. 7:**
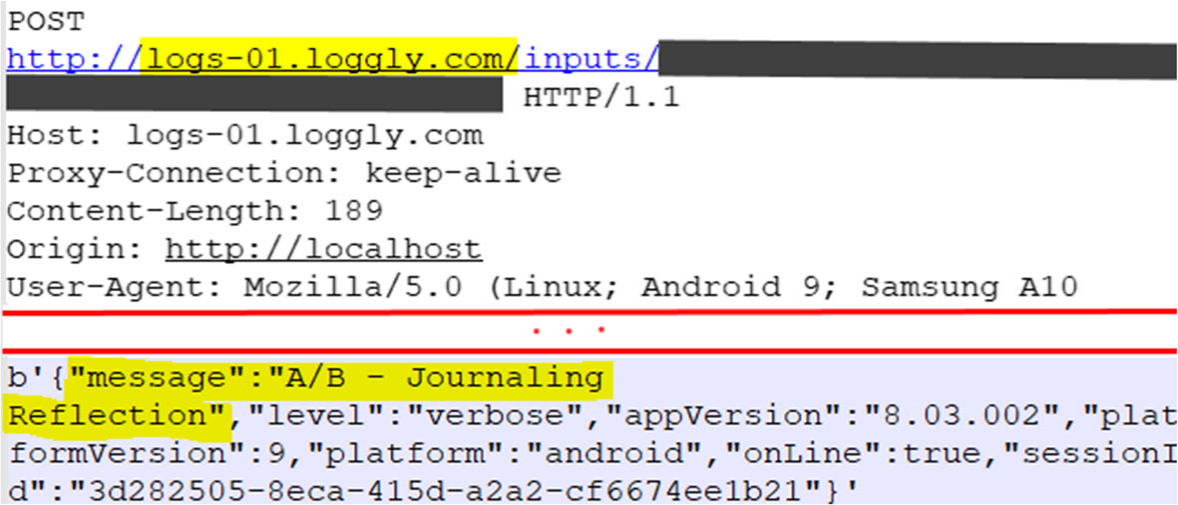
Example of 3rd-party logging service used to record apps’ activities, sending data over HTTP

Such threats are also identified during the dynamic analysis. We observed non-repudiation threats related to the disclosure of decrypted logs of network connections (NR_df7), and when a person wanting deniability cannot edit a database (NR_ds3). The analyzed apps communicate with several 3rd-parties, e.g., for marketing and advertising, cloud service provisioning, and payments services. This makes it impossible for users to determine to what extent their communication and data are collected, used, and stored. A rather worrying example is the logging of user actions in an app by a 3rd-party logging service using the insecure HTTP protocol, as shown in Fig. 7.

Table 5 shows the number of servers that the apps communicated with during the analysis. On average, an app communicated with 11.9 servers (*std* = 13.8), with a minimum of 1 and a maximum of 64 communicating servers. Most of these servers are 3rd-party service providers. On average, 81.7% (*std* = 18.3) of the servers that each app communicated were owned by 3rd-parties. Such intense use of service providers increases the risks of non-repudiation. In addition, if the data that is shared is identifiable, it will be harder to repudiate.

**Table 5:** Web servers communicated during the dynamic analysis

N. of Apps	N. of Web Servers
6	1-5
5	6-10
6	11-20
2	> 20

Table 6 presents a list of the 3rd-party domains most commonly observed in the performed analysis. App developers use such common 3rd-parties for marketing (e.g., Mixpanel, RevenueCat, Branch.io, Amplitude, Facebook), cloud service provisioning (Firebase, CrashAnalytics, Bugsnag), and payment services (e.g., Stripe and PayPal). Software developers and users often have little to no control over the data after sharing it with service providers.

**Table 6:** Most common 3rd-party domains

N. of Apps	Domain
18	google.com
15	googleapis.com
12	crashlytics.com
9	branch.io
8	facebook.com
8	gstatic.com
7	mixpanel.com
7	youtube.com
6	app-measurement.com

#### Detectability threats

Detectability refers to being able to sufficiently distinguish whether or not an IOI exists (Pfitzmann and Hansen 2010), even if the actual content is not known (i.e., no information is disclosed). Based on the detection of whether or not an IOI exists, one can infer or deduce certain information. For instance, by knowing that a user has a profile in a specific mental health service, you can deduce that they might be seeking psychological support or facing specific mental health conditions. Achieving undetectability in mobile and web applications is inherently complex, given that client-server communication is usually easily detectable.

All apps that generate network traffic present detectability threats. Threats are observed during the dynamic analysis, such as no or weak covert channel (D_df2), since data flows can be examined (D_df7) and the timing of the requests is visible (D_df13). The data stored by the apps is also detectable due to the weak access control to the data file system or database (D_df1). Software developers cannot easily address such threats, considering that existing apps would have to provide relatively advanced privacy controls, such as using covert channels and anonymous communication. The reliance on various 3rd-party service providers makes it even more challenging.

#### Disclosure of information threats

Information disclosure refers to the unwanted and unauthorised revelation of information. For data flows, the channel is insufficiently protected (e.g., un-encrypted), and the message is not kept confidential. Similarly, the information is protected with weak access control mechanisms or kept in plain text for data stored.

Threats on disclosure of information were observed in the static, dynamic, and server-side analyses, using MobSF to reverse engineer code and inspect data flows and server configuration. Based on MobSF’s static analysis, a total of 20 apps (74%) scored as Critical Risk and 4 apps (15%) as High Risk in the App Security Score. To calculate the App Security Score, MobSF first gives every app an ideal score of 100. The score changes for every finding based on its severity: high reduces 15 points, warning reduces 10 points, and good increases 5 points. If the score is greater than 100, then it is kept as 100. If the score is lower than 0, then it is kept as 10. The App Security Score falls into a final risk level, according to the ranges: Critical Risk (0 - 15), High Risk (16 - 40), Medium Risk (41 - 70), and Low Risk (71 - 100). From the beginning of the analysis, the high number of apps in Critical and High Risks suggested that many apps would have problems in terms of permissions, code vulnerabilities, trackers, etc.

**Fig. 8:**
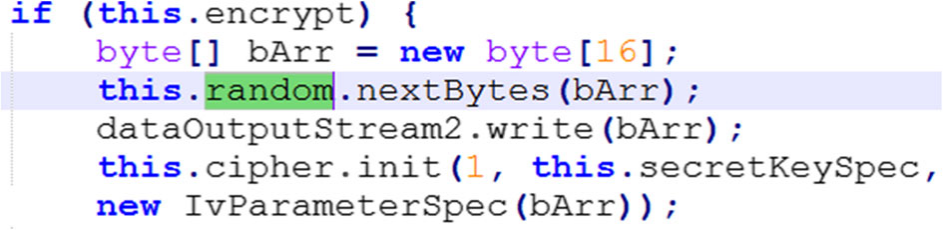
Example of insecure random (i.e., java.util.Random) used to generate IVs

**Fig. 9:**
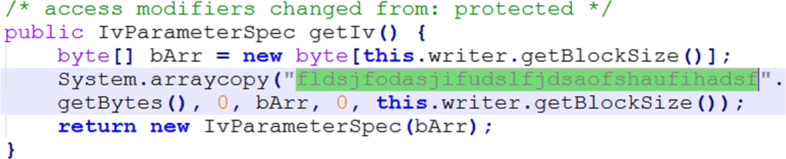
Use of hard-coded IVs

Among the prevalent types of threats, weak message confidentiality (ID_df5) was verified in several apps due to the use of insecure cryptography, which leads to no channel confidentiality (ID_df7). Manual verification of the apps’ reverse-engineered code was performed, revealing that fifteen apps used insecure PRNGs (e.g., see Fig. 8). Also, seven apps used insecure cyphers (i.e. MD5 and SHA1), and one app used an insecure cypher mode (ECB). We also manually investigated insecure Initialisation Vectors (IVs) used in the apps. IVs are used as cryptographic primitives to provide an initial state (e.g., for a cypher), and should be typically created using a cryptographic pseudorandom number generator, but sometimes an IV only needs to be unpredictable or unique. A total of 12 apps were found to have used insecure IVs. For instance, in Fig. 9, the IV is a hard-coded string of fixed value, which would weaken any resulting ciphertexts when repeatedly used for a fixed key.

**Fig. 10:**
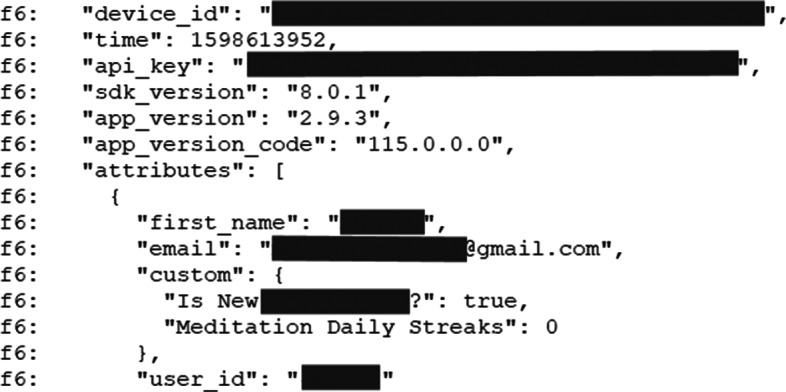
Example of sensitive information in Logcat logs

Another common threat is the lack of message confidentiality (ID_df4). During the log analysis, we sought to identify four types of data leaks, as shown in Table 7. These are alarming results as this information in Logcat logs can be accessed by other apps that are running in a device (Kotipalli and Imran 2016). Figure 10 shows an example of a Logcat log snippet identified to log personal data of the user and API keys.

**Table 7:** Analysis of Logcat logs

N. of Apps	Information disclosure issues
19	Revealing apps’ behaviour, usage, and activities.
15	Logging web traffic, parameters, Post value.
5	Revealing personal information.
4	Revealing tokens and credentials used by the app.

Threats to the stored data were also common, e.g., bypass protection scheme (ID_ds1), data intelligible (ID_ds2), or un-encrypted (ID_ds10). Only four apps have used encryption for storing files, and none have used encrypted databases. We found 15 apps that stored users’ personal information (e.g. email, password, address) in files or databases. Such information can be accessible by unintended parties (e.g., in case of device theft or malicious backups).

Disclosure of the credentials was also observed at various stages of the static and dynamic analyses. This could lead to spoofing of an external entity (S) if an attacker can obtain legitimate credentials (S_1) from an existing user (e.g., username and password) or service (e.g., API keys). For instance, when inspecting the generated network traffic, we found that 13 apps reveal API keys used to access 3rd-party services, leading to unauthorized access to micro-services and APIs. Two apps also revealed the user email and password in the HTTP header or as GET parameters. Furthermore, 18 apps stored the credentials such as passwords, tokens and keys insecurely.

#### Unawareness threats

Unawareness refers to data subjects not being aware of the impacts and consequences of sharing personal data. For instance, personal data is shared with mental health services and other services (i.e. cloud providers, analytics, advertising services). In such cases, a system itself can support users in making privacy-aware decisions. Such unawareness threats focus on a system’s provisions to guide and educate users concerning their data sharing.

Evidence of unawareness threats was observed in the static and dynamic analyses, the requests of PIAs, and the communication with developers. A type of threat concerns providing too much personal data (U_1), which can be linked to the list of permissions required by the apps to run. MobSF static analysis checks the apps for dangerous permissions, i.e., giving an app additional access to the restricted data and allowing an app to perform the restricted actions that substantially affect a system and other apps. On average, the apps have 5.6 dangerous permissions (*std* = 8.2), with apps requiring a minimum of 3 up to 30 dangerous permissions. Table 8 lists the most common dangerous permissions used by the studied apps.

**Table 8:** Most common dangerous permissions used by apps

N. of Apps	Dangerous permissions
27	android.permission.INTERNET
24	android.permission.WAKE_LOCK
23	com.google.android.finsky.permission-
	.BIND_GET_INSTALL_REFERRER_SERVICE
19	android.permission.WRITE_EXTERNAL_STORAGE
16	com.android.vending.BILLING
13	android.permission.READ_EXTERNAL_STORAGE
9	android.permission.READ_PHONE_STATE
7	android.permission.ACCESS_FINE_LOCATION
6	android.permission.RECORD_AUDIO
6	android.permission.MODIFY_AUDIO_SETTINGS
6	android.permission.CAMERA
6	android.permission.ACCESS_COARSE_LOCATION

As mentioned in Section 3.2.1, two authors manually inspected the dangerous permissions to verify whether they are necessary to serve the app’s purpose. During the evaluation, we used the apps in real mobile phones, and checked for functions that would justify the use of a given dangerous permission. Dangerous permissions that did not seem necessary were flagged and included as a potential issue in the reports later sent to developers. Most of the dangerous permissions were not deemed necessary for the apps to function. For instance, the pair of permissions READ_EXTERNAL_STORAGE and WRITE_EXTERNAL_STORAGE are not always needed, but they are dangerous because they grant an app indiscriminate access to the device’s external storage, where a user’s sensitive information may be stored. On average, the apps use 4.1 (*std* = 7.6) unnecessary dangerous permissions. Even though software developers may have justifiable purposes for requiring such permissions, users must clearly understand them.

In this study, we also took the initiative of contacting the companies whose apps were studied and requesting the PIA reports of their respective apps. This step revealed a degree of no/insufficient feedback and awareness tools (U_3), considering that PIAs reflect on the impacts of information sharing. Only three (11%) companies carried out a PIA for their apps, and only two of them made the PIA report available to us. Of the remaining companies, twenty (75%) did not answer this PIA request, and four (15%) reported not conducting a PIA. It is worth mentioning that PIAs would help companies to demonstrate compliance to data protection authorities, which relates to the following subsection on Non-compliance threats. Furthermore, if we consider mental health apps as likely to result in “high-risk” to the rights and freedoms of natural persons, PIAs are mandatory according to the EU GDPR (EU Commission 2017).

We can also consider the companies’ feedback in the responsible disclosure process. We emailed the evaluation reports consisting of all the issues found for different apps to their respective companies. We received responses from seven companies (26%) that provided us with their feedback and the actions taken (e.g., forward the reports to the technical and legal teams). The responses from software developers, lead engineers, and privacy officers were positive. They all appreciated the well-intended ethical research actions supporting them, with the desire to help build more secure and privacy-preserving apps. Three companies have reported back stating that the raised issues were or are being fixed for the subsequent releases of the apps. One company also provided a detailed response in which that company verified all the raised issues that were marked for suitable fixes. This company also asked for further feedback to check if there were any other concerns. All the communication with developers and companies was done via email.

#### Non-compliance threats

Non-compliance refers to adherence to legislation, regulations, and corporate policies. LINDDUN uses this threat category to cover privacy notices and policies that should be provided to all users to inform them about the data collected, stored, and processed by systems. Privacy policies and consent are linked, given that users have to read and understand the apps’ privacy policy to provide informed consent.

The analyses of the apps’ privacy policies, using readability scores and AI-assisted privacy tools, allowed the identification of non-compliance threats concerning incorrect or insufficient privacy policy (NC_2) and insufficient notice (NC_4). Considering the Flesch-Kincaid reading ease measurement, most apps (89%, n = 27) scored between 30-50 in the readability index, meaning that their privacy policies are difficult to read, requiring college-level education. Three apps scored a 50-60 range index, implying that the privacy policies are reasonably challenging to read, requiring 10th- to 12th-grade level education. Interestingly, only one app provided a layered privacy policy (Timpson 2009), providing a 1st-layer summary and a 2nd-layer with the complete privacy policy, making it easier to read and understand.

**Fig. 11:**
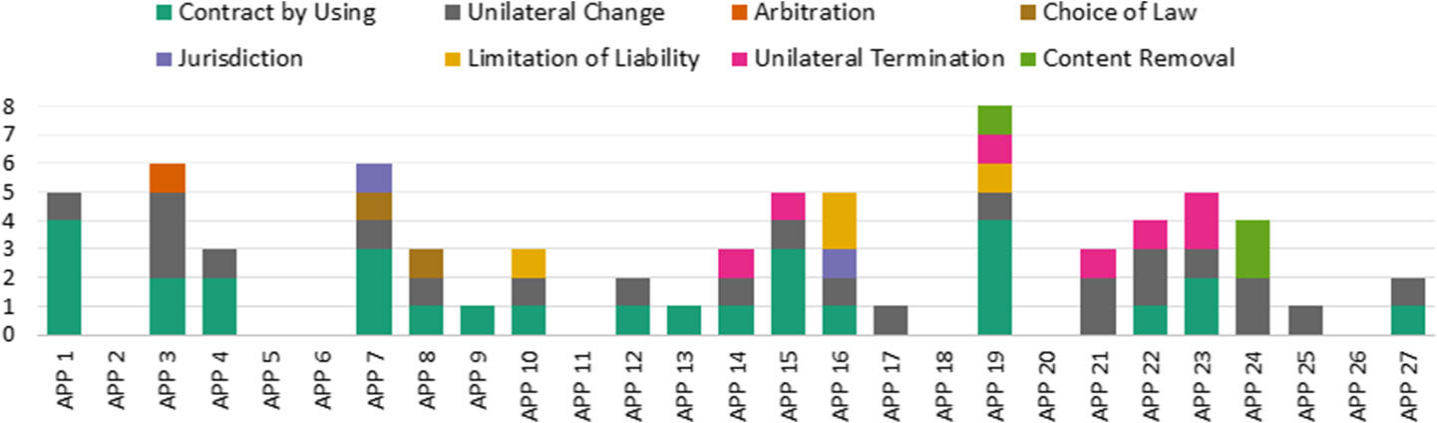
Summary of CLAUDETTE results of unfair clauses

Threats in terms of incorrect or insufficient policies (NC_2) were also revealed using the CLAUDETTE tool to identify unfair clauses. Figure 11 presents a summary of the results obtained using CLAUDETTE. On average, the apps’ privacy policies had 2,7 unfair clauses The most common type of unfair clause we observed was ‘Unilateral Change’, presented in the privacy policies of 18 apps. Furthermore, 16 privacy policies had unfair clauses in the ‘Contract by Using’ category.

We further analyzed the apps’ privacy policies using the PrivacyCheck tool, which scores the apps in terms of (1) user control over privacy and (2) GDPR compliance. We used this tool to check the privacy policies of 26 apps, except for one app that the tool failed to interpret. On average, the apps obtained a user control score of 59/100 (*std* = 15.14), and a GDPR score of 63.1/100 (*std* = 31.25).

**Fig. 12:**
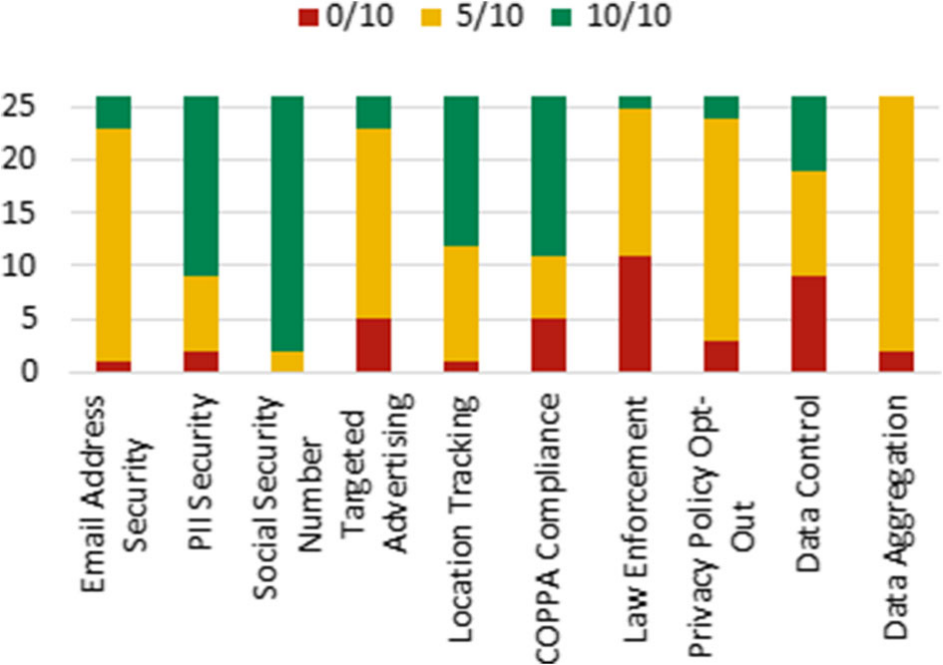
Summary of user control scores from PrivacyCheck

Figure 12 presents a more detailed summary of the PrivacyCheck scores obtained for the ten questions corresponding to the users’ control. As shown in the figure, our sample of apps scored very poorly for questions such as *“Does the site share your information with law enforcement?”* (11/26 apps scored 0/10) and *“Does the site allow you to edit or delete your information from its records?”* (9/26 apps scored 0/10). However, it appeared that the apps handled some privacy aspects more effectively, such as *“How does the site handle your Social Security number?”* (24/26 apps scored 10/10) and *“How does the site handle your credit card number and home address?”* (17/27 apps scored 10/10).

**Fig. 13:**
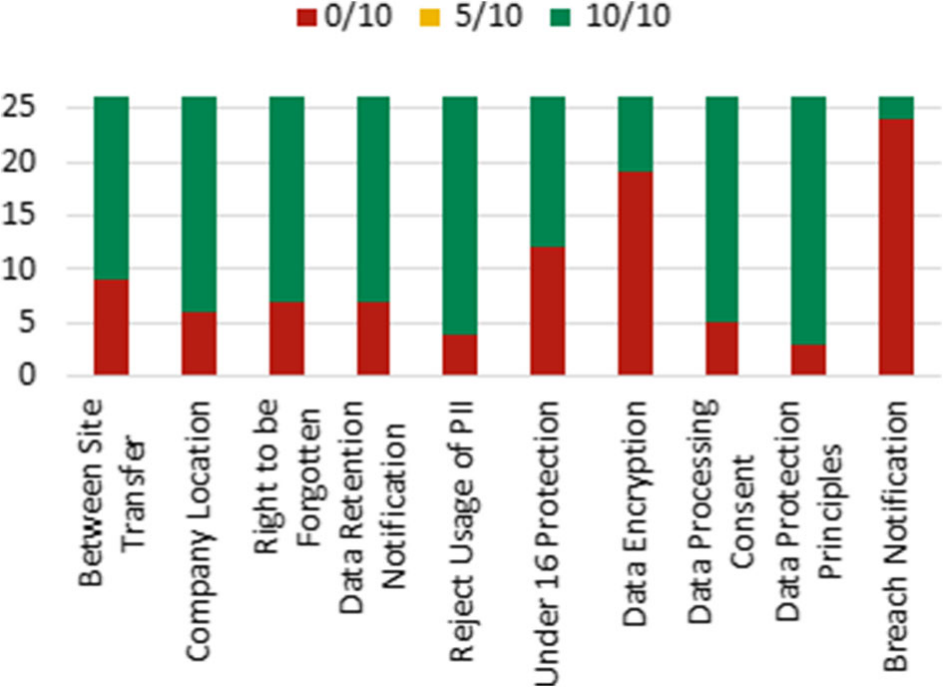
Summary of GDPR scores from PrivacyCheck

Similarly, Fig. 13 presents the PrivacyCheck scores obtained for the ten questions corresponding to GDPR compliance. The lowest compliance was observed for *“Does the site notify the supervisory authority without undue delay if a breach of data happens?”* (24/26 apps scored 0/10) and *“Does the site advise that their data is encrypted even while at rest?”* (19/26 apps scored 0/10). Most apps showed better compliance for questions such as *“Does the site implement measures that meet the principles of data protection by design and by default?”* (23/26 apps scored 10/10) and *“Does the site allow the user object to the use of their PII or limit the way that the information is utilized?”* (22/26 apps scored 10/10).

## Discussion

The study’s results enable us to answer the research question: *What is the current privacy status of top-ranked mental health apps?* Table 9 summarises the most common privacy issues and their prevalence in the studied mental health apps, contextualising findings according to the LINDDUN threat categories. Based on that, this section discusses the following concerning topics: (1) privacy impacts of mental health apps; (2) apps’ permissions and data access; (3) apps’ security testing and coding; (4) Privacy Impact Assessments and responsible disclosure; (5) privacy policies; and, (6) recommendations.

**Table 9:** Summary of main findings according to LINDDUN

Main findings	Threat examples	LINDDUN
Finding 01: Out of the 27 top-ranked mental health apps selected, most of them address the conditions of anxiety, stress, burnout and depression. Also, over a third of them address various other mental disorders, e.g., addictions, bipolar, self-harm, PTSD and OCD. Hence, these apps’ processing operations result in “high-risk” to the rights and freedoms of natural persons.	*(finding is too general to be mapped)*	$\square \square \square \square \square \square \square $
Finding 02: 74% (*n* = 20) of the apps scored as Critical Risk and 15% (*n* = 4) as High Risk in the App Security Score during the static analysis.	*(finding is too general to be mapped)*	$\square \square \square \square \square \square \square $
Finding 03: All apps require dangerous permissions to run. Our manual inspection points to an average of 4 unnecessary dangerous permissions used being used, in which read/write operations to the external storage are of primary concern.	Providing too much personal data (U_1), incorrect or insufficient privacy policies (NC_2), insufficient notice (NC_4).	$\square \square \square \square \square \blacksquare \blacksquare $
Finding 04: Manual verification of the apps’ codes shows a high prevalence of fundamental secure coding problems related to the use of insecure PRNGs (56%), insecure cyphers(56%), insecure cypher modes (26%), and insecure IVs (44%).	Weak message confidentiality (ID_df5).	$\square \square \square \square \blacksquare \square \square $
Finding 05: 96% (*n* = 26) of the apps contained hard-coded sensitive information like usernames, passwords and keys.	Spoofing an external entity (S), obtain legitimate credentials (S_1).	$\square \square \square \square \blacksquare \square \square $
Finding 06: Two apps reveal user email and credentials in the HTTP header or as GET parameters.	Spoofing an external entity (S), no message confidentiality (ID_df4), no channel confidentiality (ID_df7).	$\square \square \square \square \blacksquare \square \square $
Finding 07: Two apps made a perplexing number of HTTP(S) requests in a short period.	Linkability and identifiability of contextual data (L_df2, I_df2), disclosure of a decrypted log of network connections (NR_df7), timing requests visible (D_df13), unaware of stored data (U_2), insufficient notice (NC_4).	$\blacksquare \blacksquare \blacksquare \blacksquare \square \blacksquare \blacksquare $
Finding 08: User behaviour can also be easily extracted from web traffic logs (i.e., it is easy to perform profiling of mental health apps’ users).	Linkability and identifiability of contextual data (L_df2, I_df2), disclosure of a decrypted log of network connections (NR_df7), timing requests visible (D_df13), unaware of stored data (U_2), insufficient notice (NC_4).	$\blacksquare \blacksquare \blacksquare \blacksquare \square \blacksquare \blacksquare $
Finding 09: 68% (*n* = 13) of the apps reveal API keys used to access 3rd-party services.	Spoofing an external entity (S), obtain legitimate credentials (S_1).	$\square \square \square \square \blacksquare \square \square $
Finding 10: Most apps try to pseudo-anonymize users through and anonymous IDs or hashed advertisement IDs, but these IDs can still be used to link data among various 3rd-parties.	Linkability and identifiability of contextual data (L_df2, I_df2), person wanting deniability cannot edit database (NR_ds3), timing requests visible (D_df13), unaware of stored data (U_2), insufficient notice (NC_4).	$\blacksquare \blacksquare \blacksquare \blacksquare \square \blacksquare \blacksquare $
Finding 11: Apps communicate with a large number of 3rd-parties, for marketing and advertising, cloud service pro-visioning, and payments services.	Person wanting deniability cannot edit database (NR_ds3), unaware of stored data (U_2), insufficient notice (NC_4).	$\square \square \blacksquare \square \square \blacksquare \blacksquare $
Finding 12: All analyzed apps reveal users’ usage and apps’ behaviour in the Android system logs (i.e., Logcat), which is visible to all applications in the system.	Weak access control to data (base) (I_ds1), bypass protection scheme (ID_ds1), data intelligible (ID_ds2).	$\square \blacksquare \square \square \blacksquare \square \square $
Finding 13: 79% (*n* = 15) of the apps store data in plain-text in the file system or in databases.	Weak access control to data (base) (I_ds1), unencrypted (ID_ds10).	$\square \blacksquare \square \square \blacksquare \square \square $
Finding 14: 95% (*n* = 18) of the apps reveal some credentials (e.g., API keys and tokens) in the stored data.	Weak access control to data (base) (I_ds1), obtain legitimate credentials (S_1).	$\square \blacksquare \square \square \blacksquare \square \square $
Finding 15: 79% (*n* = 15) of the apps’ databases are not encrypted.	Weak access control to data(base) (I_ds1), unencrypted (ID_ds10).	$\square \blacksquare \square \square \blacksquare \square \square $
Finding 16: Twenty apps (75%) did not report whether they conducted or not a PIA, and 4 (15%) apps explicitly declared not conducting a PIA.	No/insufficient feedback and awareness tools (U_3), insufficient notice (NC_4).	$\square \square \square \square \square \blacksquare \blacksquare $
Finding 17: Flesch-Kincaid Reading Ease average is 42 (i.e., Difficult to read) for the cohesiveness and complexity of the apps’ privacy policies.	Incorrect or insufficient privacy policy (NC_2), insufficient notice (NC_4).	$\square \square \square \square \square \square \blacksquare $
Finding 18: An average of 2,7 unfair clauses was revealed for the analyzed privacy policies.	Incorrect or insufficient privacy policy (NC_2), insufficient notice (NC_4).	$\square \square \square \square \square \square \blacksquare $
Finding 19: The average user control score that the apps obtained was 59/100 (*std* = 15.14), and the average GDPR score was 63.1/100 (*std* = 31.25).	Incorrect or insufficient privacy policy (NC_2), insufficient notice (NC_4).	$\square \square \square \square \square \square \blacksquare $
Finding 20: 74% (*n* = 20) of the companies have not replied to the reports sent for responsible disclosure.	No/insufficient feedback and awareness tools (U_3), insufficient notice (NC_4).	$\square \square \square \square \square \blacksquare \blacksquare $

### Privacy impacts of mental health Apps

Even though mental health apps have higher privacy impacts, the results show that these apps contain most of the privacy and security issues found in an average Android app. For example, our analysis identified vulnerabilities related to all seven Android app vulnerability categories (i.e., cryptography API, inter-component communication, networking, permission, data storage, system processes, and web API) presented by Ranganath and Mitra (2020). Furthermore, various privacy issues were identified, such as insufficient levels of information handling, similar to what other researchers have observed in different types of mobile apps (Huckvale et al. 2019; Powell et al. 2018).

Privacy violations in mental health apps tend to have severe negative impacts on the rights and freedoms of natural persons, therefore calling for higher levels of protection and safeguards. Some issues identified in this privacy analysis would have a lower impact in a general Android app (e.g., WhatsApp, Twitter, Netflix apps). For example, disclosure of identifiers to 3rd-parties, such as IMEI, UUID and IP address, would have a low impact in a general app. Perhaps, most users would not even consider it as an issue. In contrast, mental health app users would consider this invasive since most users would not even want other people to know that they are using mental health apps. Research has shown that breaches of mental health information have severe repercussions, such as exploitative targeted advertising and negative impacts on an individual’s employability, credit rating, or ability to access rental housing (Parker et al. 2019).

### Apps’ permissions and data access

During the static analysis, we found that all apps use one or more dangerous permissions. Many of these permissions could be avoided or at least better explained to end-users. For instance, the pair of dangerous permissions READ_EXTERNAL_STORAGE and WRITE_EXTERNAL_STORAGE. Based on our manual analysis of apps’ permissions (Section 4.2.6), we noticed that the apps rarely need access to external storage. Thus, these permissions could have been avoided or more carefully used.

The apps also request such permissions (i.e., get user approval) when they are first opened. Users can indeed revoke dangerous permissions from any app at any time, provided that they know how to change the configurations. However, it would be recommended that app developers ask for permissions “in context”, i.e., when the user starts to interact with the feature that requires it. Also, if permissions are not essential for the apps to function, they could be disabled by default, i.e., running the app most privately. Whilst it appears that the apps are becoming greedier about users’ data, there are also flaws in the Android permission system that should to be considered. As discussed in Alepis and Patsakis (2017), Android still does not allow users to have full access to an app’s permissions so that users can revoke access to both normal and dangerous permissions individually. The system automatically grants the normal permissions, and the users have little to no transparency about them. The dangerous permissions are granted as a group, i.e., the entire permission group CONTACTS is granted, including permissions GET_CONTACTS, READ_CONTACTS, and WRITE_CONTACTS, instead of letting users grant or deny them separately. Alepis and Patsakis (2017) also stress that even normal permissions can lead to user profiling or leaks of sensitive information (e.g., use GET_PACKAGE_SIZE to list all the user’s installed apps), or have the potential for accidental or malicious misuse (e.g., use INTERNET to open network sockets just to fetch ads). However, we assert that it should be the developers’ responsibility to understand the Android’s permission system and appropriately use it in a privacy-preserving manner.

Future research could also focus on the apps’ permissions, data access, and sharing behaviours over more extended periods. For instance, similar to Momen (2020), in which researchers had apps installed on real devices over time (e.g., months) analyzing the apps’ behaviour under various conditions. Ideally, developers would benefit the most if they could rely on a testbed for privacy assessment, as the one proposed by the REsearch centre on Privacy, Harm Reduction and Adversarial INfluence online (REPHRAIN) (Gardiner et al. 2021). Such testbed would enable developers to only drop their app file into an user interface, following a wizard-based tool. The testbed then runs multiple static and dynamic privacy tests against the file and produces a report in a comma-separated format, which the developer can download for their own analysis.

### Apps’ security testing & coding

The results from our static analysis (Section 4.2.5) showed that most apps are at critical risk (*n* = 20) or high risk (*n* = 4). Vulnerabilities such as hard-coded secrets (Lee 2019), use of weak algorithms and protocols (ECB, TLS 1.0, etc.), weak IVs, and insecure PRNGs (Egele et al. 2013) were also verified. The MobSF tool has been continuously upgraded, making such security testing relatively straightforward. App developers could have identified most of these issues using MobSF’s automated static analysis. The prevalence of such vulnerabilities suggests that app developers are not adhering to the basic principles of secure coding. Furthermore, it is worth stressing that many of our findings were identified in the dynamic analysis. The inspection of network traffic, stored data, and logs can reveal several issues that a static analysis alone cannot.

A recent study found that 85% of mHealth developers reported little to no budget for security (Aljedaani et al. 2020) and that 80% of the mHealth developers reported having insufficient security knowledge (Aljedaani et al. 2020; Aljedaani et al. 2021). We believe that the developers of the mHealth apps analyzed in this study faced similar challenges that are also evident from the following observations concerning secure coding/programming. First, the use of insecure randoms, cypher modes, and IVs, i.e., incorrect use of cryptographic components. Second, the insecure logs, leaking the app’s behaviour and the user’s data, either internally to the system logs (e.g., Logcat) or externally to cloud-based logging services (e.g. Loggly). Third, the presence of hard-coded information, such as tokens and API keys. Such findings signal that app developers require more security training and that security testing may not be part of the development process. Besides, developers can also benefit from existing plugins for integrated development environments, such as CogniCrypt (Krüger et al. 2017) for Eclipse, that generates secure implementations for common programming tasks that involve cryptography and alerts for misuses of cryptographic APIs.

### PIAs, DPIAs, and responsible disclosure

From the start, we contacted all the relevant companies whose apps we selected for study for obtaining the PIAs reports (if available). However, we received only two public PIA reports. These PIA reports were relatively brief, lacking sufficient information about the apps’ systems and components. PIAs should usually start with a high-level data flow diagram that shows what personal data is collected and how it is processed and shared among 3rd-party services (EU Commission 2017). We assert that it is important for an mHealth app to identify the potential privacy threats and apply suitable countermeasures for eliminating or mitigating the identified risks during appropriate phases of development/evolution. As per our findings, a large majority of the mHealth apps developers seem to be unaware of the PIA requirements that are usually mandatory according to some regulations, such as GDPR.

Whilst it is understandable that performing and updating full-fledged PIAs is a time-consuming process, e.g., see the PIA (Iwaya et al. 2019), mHealth apps development companies and developers can still perform minimal PIAs. An example comes from the proposal of a code of conduct for mHealth privacy by Mantovani et al. (2017) and their recommended PIA questionnaire. The knowledge and time invested in performing a PIA and making that public will help increase the trust of the end users and the relevant authorities.

We also contacted the companies during the responsible disclosure process for providing them with a list of found issues for each app. However, the current study shows that most of these companies offered little to no response on the privacy concerns. Although seven companies (26%) replied acknowledging the received reports, there were only three companies that reported back stating that they would address the raised issues. Only one company provided a detailed account, verifying all the raised issues and proposing fixes. Such a lack of answers indicates a troubling situation in which it is difficult to discern whether or not the mHealth apps development companies will pay due attention to address privacy issues.

### Privacy policies: transparency, consent and intervenability

All the analyzed mHealth apps had a privacy policy. This is quite positive if compared to other studies that reported that only 46% of dementia apps (Rosenfeld et al. 2017) and 19% of diabetes apps (Blenner et al. 2016) had a privacy policy. This is likely because we analyzed only top-ranked apps with large user bases. However, the readability scores of the privacy policies are still low. According to other studies, the average grade-level readability should be calculated as the average of the scores from the Gunning Fog, Flesh-Kincaid Grade Level, and SMOG formulas (Robillard et al. 2019; Sunyaev et al. 2014). In such case, the average grade-level readability for the analyzed privacy policies was 13.21, consistent with the scores of 13.78 in Robillard et al. (2019) and 16.00 in Sunyaev et al. (2014). Privacy policies are still hard to read, raising concerns with regards to transparency and consent.

Privacy policies also present unfair clauses, of which “contract by using” and “unilateral change” are the two most common types. Contract by using is incredibly unfair in the case of mHealth apps. Such apps should rely on explicit informed consent since they handle sensitive personal data of people who may be considered to be in a more vulnerable and fragile state. The EU GDPR (Art. 4 (11) defines consent as freely given, specific, informed and with explicit indication of the data subject’s wishes to use the system and have his or her data collected and processed (European Commission 2016). Contract by using defies this idea of consent. Companies should review their apps’ privacy policy and, most importantly, change the apps to honestly inform users, recording their consent to collect and process data.

Most apps’ consent process was just an initial screen presenting the privacy policy and an “I agree” button. Understandably, developers design their apps with as few steps as possible in the onboarding process, aiming to reduce friction and improve users’ experience. However, poor privacy also causes a bad user experience. Balancing privacy and user experience is challenging and demands further investigation. However, developers could ask themselves: *“Would my users be surprised if they knew about all the data that is collected, the processing purposes, or the extent of data sharing?”* Any privacy “surprises” reveal issues that need to be raised and discussed, users should be informed, and the system’s design should be reviewed.

For instance, many mHealth apps rely on advertising as monetary revenue. Users of mHealth apps, even if de-identified, are still targeted with personalised advertisements based on their unique “anonymous” IDs (e.g., uuid and aaid). Also, the advanced paradigms of personal advertising, such as cross-device tracking (CDT), are commonly used to monitor users’ browsing on multiple devices and screens for delivering (re-)targeted ads on the most appropriate screen. For instance, if a person downloads an mHealth app on one’s mobile device, it is likely that person will see other ads about mental health in one’s Facebook timeline when using a PC. Researchers have already found that CDT undoubtedly infringes users’ online privacy and minimizes their anonymity (Solomos et al. 2019). Besides, there is a risk of exploitative advertising to individuals who may be vulnerable due to mental health conditions. Such extent of data processing is likely to surprise users (and developers), unaware of privacy risks and impacts. These observations enable us to support the growing arguments that apps development is intrinsically linked to the online advertising businesses, which may give little to no control on the management and utilization of data to those from whom the data is gathered, i.e., end users.

## Limitations

Some limitations in terms of the methodology need to be considered when interpreting the findings of this study. It should be noted that today there is a myriad of other open-source tools for penetration testing that can be used for studies like this one. For example, static analyzers, such as FlowDroid (Arzt et al. 2014), Amandroid (Wei et al. 2018) or RAICC (Samhi et al. 2021), and dynamic analyzers, such as IntelliDroid (Wong and Lie 2016) and TaintDroid (Enck et al. 2014). For planning future studies, we would encourage the researchers to seek more resources that would enable them to select and further review other app categories. However, it may not be possible for researchers doing empirical studies to consider all sorts of tools available due to scope and resources limitations. In this study, apart from tools such as Drozer, Qualys SSL, CLAUDETTE and PrivacyCheck, we relied on MobSF for various static and dynamic analyses since this framework integrates many tools to provide a broad coverage of penetration tests. The community around MobSF also provides its users with free support channels, learning materials, and a straightforward installation and setup process. For such reasons, MobSF is widely used in academia and industry.

To identify the mental health apps on Google Play Store, we used the google-play-scraper to automate the search process. This tool required us to set a specific location for the search, which we defined as Australia. Even though most of the investigated apps come from the regions of North America and Europe, it is important to consider that apps available in Australia might not be representative of the overall Android mHealth apps ecosystem.

We also manually investigated the code snippets flagged for insecure PRNGs, cyphers, and cypher modes during the static analysis. That is, we limited our analysis to the files flagged by MobSF. However, we observed that some of the reported code snippets used insecure PRNGs and cyphers to create wrapper classes and util methods for the original functionality. Even though using these wrapper classes and util methods in security contexts would lead to a security vulnerability, our analysis did not investigate such usages as it would increase the complexity and resources required for the study. We have shared this observation with the studied apps’ development teams as part of the responsible disclosure process with the suggestion to take these points into consideration while reading our findings.

During the dynamic analysis, some apps were not compatible to run on the Genymotion emulator with MobSF. Hence, the results are limited to a smaller sample of 19 apps that were fully dynamically analyzed. This process required the manual operation of the apps, attempting to cover all of the accessible functionalities. However, we neither performed any credit card payments nor paid to test the premium features, limiting the extent of testing.

Regarding the analysis of the privacy policies, we relied on two AI-based tools: (1) CLAUDETTE, to identify unfair clauses; and, (2) PrivacyCheck, to calculate user’s control and GDPR compliance scores. Although such tools give us a metric for comparison, an ideal analysis of privacy policies would require a legal analysis of the text made by a privacy lawyer. These AI-based tools also have some limitations concerning their accuracy. According to the creators of these tools, CLAUDETTE has an accuracy of 78% for identifying unfair clauses and an accuracy between 74%-95% for distinguishing between unfair clause categories (Lippi et al. 2019). PrivacyCheck has an accuracy of 60% when scoring privacy policies for the ten user control questions and the ten GDPR questions (Zaeem et al. 2020). Thus, the results should be interpreted with such limitations in mind.

## Conclusion

Mental health apps offer new pathways for people to seek psychological support anywhere and anytime. The innovative use of technological advances in mobile devices for providing mental health (or well-being) support purports to significantly improve people’s quality of life. However, the mobile apps are increasingly vulnerable to data privacy breaches as a result of security attacks. A data privacy breach of an app may result in financial, social, physical or mental stress. Given the users of mental health apps are usually facing psychological issues such as depression, anxiety and stress, the detrimental impact of an app’s data privacy breach can have more significant negative impact on users. Thus, it is of utmost importance that the development of such mHealth apps follows the practices that ensure privacy by design.

We decided to empirically study the data privacy of mental health apps. Our empirical investigation shows a high prevalence of information disclosure threats, mainly originating from insecure programming. Threats related to linkability, identifiability, non-repudiation and detectability are also exacerbated by the large number of 3rd-parties in the apps’ ecosystem, facilitating profiling of users and exploitative advertising. Apps also lack transparency and sufficient notice mechanisms, leading to unawareness and non-compliance threats.

This study has provided us with sufficient empirical evidence to assert that mHealth apps in general and also mental health apps in particular ought to be developed by following a privacy by design paradigm (Cavoukian et al. 2009; Gürses et al. 2011). Moreover, this study has also enabled us to surmise that apart from developers, other stakeholders can also play important roles in ensuring data privacy in mHealth apps. Based on this research, we have compiled a list of data-informed actionable measures as a set of recommendations for ensuring data privacy in mHealth apps. Table 10 provide the list of recommendations linked to the findings presented in Table 9. We expect that these recommendations will enable all the key stakeholders, particularly the apps developers, to play their respective parts in order to ensure the privacy of the data of mHealth apps.

**Table 10:** Summary of recommendations to multiple stakeholders

**Organizations**	**Find.**
→ **Undertake a Privacy Impact Assessment** – Demonstrate compliance by conducting Privacy Impact Assessment, even if not a full-fledged PIA. There are more concise/simplified methodologies for mHealth.	16
→ **Assume Mental Health Apps as High-Risk Systems** – Development processes should be fine-tuned to give better emphasis to security and privacy. When developing a health app (or mental health app), higher levels of security and privacy should be considered compared to other general apps.	1, 8
→ **Engage with Experts** – Better engagement of security and privacy experts in the development and evaluation, as well as in the writing of privacy policies to avoid unfair clauses.	20
→ **Write Readable Privacy Policies** – Enhance transparency and openness by writing accessible Privacy Policies that truly allow users to understand and make informed decisions.	17
**Software developers**	**Find.**
→ **Beware of the Unskilled and Unaware** – Likely, app developers do not know the extent of security and privacy risks of using 3rd-party SDKs and APIs. That, matched with the lack of security knowledge, might make them prone to a Dunning-Kruger effect on security knowledge, i.e., overseeing and underestimating security and privacy issues while also overestimating their levels of secure coding abilities (Ament 2017; Wagner and Mesbah 2019).	4, 5, 6, 9, 12, 13, 14, 15
→ **Connect the Privacy Policy to the System’s Design** – Even though privacy policies are not within the software developers responsibility, they should be familiar with their app’s privacy policy and terms of service. Interact with lawyers (or whoever is responsible for writing and updating the privacy policy) whenever necessary to correct information on data collection, purpose limitation and specification, and ensure security and privacy by design.	17, 18, 19
→ **Engineer Privacy By Design and By Default** (Art. 25 GDPR (European Commission 2016)) – Software developers should be aware that the GDPR states that *“controller shall implement appropriate technical and organisational measures”*. Even though implementing the “state-of-the-art” is not always “technically” possible in all organisations and systems, vulnerabilities related to very basic secure coding practices are rather concerning.	2, 4, 7, 9, 13, 14, 15
→ **Collect Valid Consent with Responsible On-boarding** – Even though the use of proper consent mechanisms may add friction to the on-boarding process, mental health apps rely on user consent to operate, so it is important that valid consent is being collected. After consent, apps should operate in the most privacy-preserving way by default (e.g., no advertising), and the consent withdrawal should be as easy as providing consent.	8, 10, 11, 18
**End-users and Health Practitioners**	**Find.**
→ **Stand Up for Your Rights** – Users that value their privacy can exercise rights by requesting more privacy-friendly apps. Users can question the current privacy policies and consent mechanisms. Request access to their data and better information on the nature of the data processing.	10, 11, 16, 19
→ **Recommend Reputable Apps for Mental Health** – Health practitioners should encourage their patients to take higher control over their treatment and journey towards better mental health. Mental health apps can help with that, but practitioners should pay careful attention and recommend only apps that respect users’ privacy.	16, 19
**Mobile App Distribution Platforms**	**Find**
→ **Raise the Bar for High-Risk Apps** – App distributors could require better privacy measurements to be put in place. Distributors could also categorise high-risk apps, adding filters for health genre apps.	1, 16
→ **Enhance Trust and Transparency** (Bal and Rannenberg 2014) – App distributors could also add useful privacy information about apps, especially about privacy consequences to support decision-making, and add privacy ratings for apps based on their data-access profiles and purposes of data access.	8, 10, 11, 12
**Smartphone Platform Providers**	**Find.**
→ **Call for Privacy-Friendly System Apps and API Frameworks** (Bal and Rannenberg 2014) – Smartphone providers could develop common systems to keep track of sensitive information flows, as well as to communicate observed behaviour to users, and provide developers with standardised ways to explain permission requests.	3

These recommendations also serve to reiterate the fact that developers alone cannot implement all the safeguards to mitigate, reduce or eliminate the identified threats. The companies’ leaders and top management are the ones who define the business models around the mental health apps. For instance, when considering the excessive use of 3rd-parties and data brokers, the software developers might be able to raise privacy issues, but it is ultimately the responsibility of the leaders to re-think and adopt more privacy-preserving business strategies. Simply put, no amount of technical and organisational privacy controls can fix a broken business model that inherently undermines people’s privacy.

This empirical study suggests that companies and app developers still need to be more knowledgeable and experienced when considering and addressing privacy risks in the app development process. At the same time, leaders and managers need to review their business models and re-think their design practices in the organisations. Raising awareness among users and health professionals is also crucial. Users should drive the demand for more privacy-preserving apps. Mental health professionals should carefully evaluate the apps to recommend privacy-friendly and safe apps to their clients.

Besides, there are also initiatives that the app distribution platforms (e.g., Google Play Store) and the smartphone platform providers (e.g., Android) can take to enhance privacy in the ecosystem. App stores can increase the vetting process for high-risk apps, such as those in medical, health and fitness application categories. Also, as suggested by (Bal and Rannenberg 2014), the app stores can provide more helpful privacy information about the apps (e.g., using privacy rating scale), and smartphone platforms can provide privacy-enhancing mechanisms in the operational systems.

## Electronic supplementary material

Below is the link to the electronic supplementary material.

(xlsx 76.3 KB)

## Data Availability

Part of the data generated and analyzed during this study is included in this published article and as supplementary material in the file “Mapping Apps’ Issues to LINDDUN”. Other datasets generated and analyzed during this study are not publicly available in order to maintain the studied apps de-identified.

## Footnotes

1 The google-play-scraper is a Node.js module to scrape application data from the Google Play store. Website: https://www.npmjs.com/package/google-play-scraper

2 Mobile Security Framework (MobSF) is an automated, all-in-one mobile application (Android/iOS/ Windows) pen-testing, malware analysis and security assessment framework capable of performing static and dynamic analysis. Website: https://mobsf.github.io/Mobile-Security-Framework-MobSF/

3 Android’s documentation for the class Manifest.permission. Website: https://developer.android.com/reference/android/Manifest.permission

4 Genymotion is an emulator for Android devices. Website: https://www.genymotion.com/

5 Drozer offers a comprehensive security and attack framework for Android. Website: https://labs.f-secure.com/tools/drozer/

6 Database browser for SQLite databases. Website: https://sqlitebrowser.org/

7 Qualys SSL is a free online service to perform a deep analysis of the configuration of any SSL web server on the public Internet. Website: https://www.ssllabs.com/ssltest/

8 The WebFX Readability Test Tool provides a way to test the readability of any textual content. Website: https://www.webfx.com/tools/read-able

## Declarations

The work has been supported by the Cyber Security Cooperative Research Centre (CSCRC) Limited, whose activities are partially funded by the Australian Government’s Cooperative Research Centres Programme. The work of L.H. Iwaya has also been partially supported by the European Commission’s H2020 Programme via the CyberSec4Europe project (Grant: 830929), the Swedish Knowledge Foundation via the TRUEdig project, and the Region Värmland via the DigitalWell Arena project (Grant: RV2018-678). Awais Rashid is supported by REPHRAIN, National Research Centre on Privacy, Harm Reduction and Adversarial Influence Online (EPSRC Grant: EP/V011189/1).
